# A burst of ABC genes in the genome of the polyphagous spider mite *Tetranychus urticae*

**DOI:** 10.1186/1471-2164-14-317

**Published:** 2013-05-10

**Authors:** Wannes Dermauw, Edward John Osborne, Richard M Clark, Miodrag Grbić, Luc Tirry, Thomas Van Leeuwen

**Affiliations:** 1 Department of Crop Protection, Faculty of Bioscience Engineering, Ghent University, Ghent B-9000, Belgium; 2 Department of Biology, University of Utah, Salt Lake City, Utah 84112, USA; 3 Center for Cell and Genome Science, University of Utah, Salt Lake City, Utah 84112, USA; 4 Department of Biology, The University of Western Ontario, London N6A 5B7, Canada; 5 Instituto de Ciencias de la Vid y del Vino CSIC, Universidad de la Rioja, Logroño 26006, Spain

**Keywords:** Acari, RNA-seq, Microarray, Phase III detoxification, Duplication, Major facilitator superfamily

## Abstract

**Background:**

The ABC (ATP-binding cassette) gene superfamily is widespread across all living species. The majority of ABC genes encode ABC transporters, which are membrane-spanning proteins capable of transferring substrates across biological membranes by hydrolyzing ATP. Although ABC transporters have often been associated with resistance to drugs and toxic compounds, within the Arthropoda ABC gene families have only been characterized in detail in several insects and a crustacean. In this study, we report a genome-wide survey and expression analysis of the ABC gene superfamily in the spider mite, *Tetranychus urticae*, a chelicerate ~ 450 million years diverged from other Arthropod lineages. *T*. *urticae* is a major agricultural pest, and is among of the most polyphagous arthropod herbivores known. The species resists a staggering array of toxic plant secondary metabolites, and has developed resistance to all major classes of pesticides in use for its control.

**Results:**

We identified 103 ABC genes in the *T*. *urticae* genome, the highest number discovered in a metazoan species to date. Within the *T*. *urticae* ABC gene set, all members of the eight currently described subfamilies (A to H) were detected. A phylogenetic analysis revealed that the high number of ABC genes in *T*. *urticae* is due primarily to lineage-specific expansions of ABC genes within the ABCC, ABCG and ABCH subfamilies. In particular, the ABCC subfamily harbors the highest number of *T*. *urticae* ABC genes (39). In a comparative genomic analysis, we found clear orthologous relationships between a subset of *T*. *urticae* ABC proteins and ABC proteins in both vertebrates and invertebrates known to be involved in fundamental cellular processes. These included members of the ABCB-half transporters, and the ABCD, ABCE and ABCF families. Furthermore, one-to-one orthologues could be distinguished between *T*. *urticae* proteins and human ABCC10, ABCG5 and ABCG8, the *Drosophila melanogaster* sulfonylurea receptor and ecdysone-regulated transporter E23. Finally, expression profiling revealed that ABC genes in the ABCC, ABCG ABCH subfamilies were differentially expressed in multi-pesticide resistant mite strains and/or in mites transferred to challenging (toxic) host plants.

**Conclusions:**

In this study we present the first comprehensive analysis of ABC genes in a polyphagous arthropod herbivore. We demonstrate that the broad plant host range and high levels of pesticide resistance in *T*. *urticae* are associated with lineage-specific expansions of ABC genes, many of which respond transcriptionally to xenobiotic exposure. This ABC catalogue will serve as a basis for future biochemical and toxicological studies. Obtaining functional evidence that these ABC subfamilies contribute to xenobiotic tolerance should be the priority of future research.

## Background

ATP-binding cassette (ABC) proteins form one of the largest protein families that are present in all living organisms on earth. The majority of ABC proteins are membrane bound primary transporters, using ATP to translocate substrates across extra- and intracellular membranes. In addition, these ABC transporters are mostly uniporters, mediating the unidirectional translocation of a substrate [1-4]. The Major Facilitator Superfamily (MFS) is another large transporter family present in all living organisms, but as opposed to ABC transporters, it comprises secondary carriers that can be either uniporters, symporters or antiporters [5]. In most ABC proteins two types of domains can be distinguished, an ATP-binding domain (also named the nucleotide binding domain (NBD)) and a transmembrane domain (TMD). The highly conserved NBD contains three motifs: a Walker A and Walker B domain and the ABC signature (LSSG-motif). The NBD binds and hydrolyses ATP and provides energy to transport substrates. The TMD consists of five to six membrane spanning helices and provides the specificity for the substrate. Full transporters comprise two NBDs and two TMDs while half transporters have only one of each type and require homo- or heterodimerization to form a functional unit [1-4]. Based on the homology of their NBDs, ABC proteins have been divided into seven subfamilies, ABCA to ABCH [1,6]. Interestingly, the ABCH subfamily was discovered during analysis of the *Drosophila melanogaster* genome and is present in all sequenced arthropod genomes to date and teleost fish, but not in mammals, plants or fungi [6-16].

In humans, ABC proteins mainly function in the membrane transport of substrates, including amino acids, sugars, lipids, inorganic ions, polysaccharides, metals, peptides, toxic metabolites and drugs [2,4]. In addition to transporters, the human ABC protein superfamily also contains ion channels (CFTR), receptors (SUR1 and 2) and proteins involved in translation (human ABCE and ABCF1, 2 and 3) [1]. Mutations in ABC genes have been linked to several human disorders, like cystic fibrosis, adrenoleukodystrophy, sitosterolemia and diabetes [17,18]. Furthermore, within the ABCB, C and G subfamilies, many genes code for proteins that contribute to resistance of cancer cells against chemotherapeutic agents: the multidrug resistance proteins or P-glycoproteins (MDR or P-gps, members of the ABCB subfamily), the multidrug resistance-associated proteins (MRP, members of the ABCC subfamily) and the breast cancer protein (BCRP or human ABCG2) [19,20]. In insects, it has been shown that ABC transporters have functions that affect metabolism, development and resistance to xenobiotics including insecticides and plant secondary toxic compounds (allelochemicals) [14]. Some ABC transporters have specific functions that are well documented in arthropods. In *D*. *melanogaster* the ABCC transporter Mdr49 controls the export of a germ cell attractant [21]. *D*. *melanogaster* white, on the other hand, is a member of the ABCG subfamily and is involved in the uptake of pigment precursors in the developing eye [22]. Its orthologs in *Bombyx mori* (Bmwh3) and *T*. *castaneum* (TcABCG-9B) have similar functions, and *w*-*3*^*oe*^*B*. *mori* mutants and *TcABCG*-*9B* dsRNA injected adult beetles have white eyes [8,23]. In the tobacco hornworm, *Manduca sexta*, orthologs of human P-gps are essential as they prevent the influx of nicotine across the blood brain barrier [24,25]. Insect orthologs of human P-gps and MRPs have also been frequently linked to pesticide resistance [26-29]. Resistance to pesticides in insects is either related to reduced target-site sensitivity or sequestration/metabolism of the pesticide before it reaches the target site by quantitative or qualitative changes of genes involved in the detoxification process [30,31]. These “detoxification” genes comprise members of the P450 mono-oxygenases (P450s), glutathione-S-transferases (GSTs), carboxyl/cholinesterases (CCEs) and also the less known ABC transporters. Although the ABC transporters have often been overlooked in studies that describe the detoxification toolkit in sequenced insect genomes [32-34], clear examples of their importance in detoxification have been documented. For example, Lanning et al. [28] showed that increased expression of human P-gp orthologues in *H*. *virescens* was associated with resistance to thiodicarb, and a mutation in the same ABCC member of four different lepidopteran species was recently associated with resistance to the Cry1A toxin [27,29].

Complete and correctly annotated gene inventories are a prerequisite to study the biological role and evolutionary history of ABC genes. Among arthropods, detailed studies of ABC families have been published for members of several different insect orders [1,8,9,12,14,16] and the crustacean *Daphnia pulex*[11]. In contrast, besides the identification of nine ABC genes in the mange mite *Sarcoptes scabiei*[35], there are no reported studies in the subphylum Chelicerata (spiders, scorpions, mites and ticks), one of the most diverse groups of terrestrial animals [36]. Recently, the first published draft genome sequence of a chelicerate, the two-spotted spider mite, *Tetranychus urticae*, was reported [37]. The spider mite is one of the most polyphagous herbivores known, and has been documented to feed on more than 1,100 plant species that belong to more than 140 different plant families, including many that produce toxic compounds [38,39]. In addition, spider mites are major agricultural pests and are the “resistance champion” among arthropods as they have the most documented instances of resistance to diverse pesticides [31,40]. The molecular mechanisms underlying spider mite resistance to xenobiotics (pesticides and allelochemicals) are less understood compared to insects [31,41]. However, the availability of the draft genome sequence now provides unique information and tools for the study of the role of gene families involved in xenobiotic metabolism in spider mites [42-44]. Characterization of spider mite gene families associated with detoxification of xenobiotics, including ABC genes, is the first step towards a better understanding of how spider mites cope with these compounds [44]. An initial preliminary analysis of ABC genes in the spider mite genome focused solely on ABCB and ABCC subfamilies [37], and lacked a complete description of the phylogenetic relationships with other metazoan ABCs. In this study, we provide a detailed comparison of all ABC subfamilies (ABCA-ABCH) in *T*. *urticae* with those of the insect *D*. *melanogaster*, the crustacean *D*. *pulex*, the nematode *Caenorhabditis elegans* and the mammal *Homo sapiens*. Further, we show that expression levels of ABC genes change in pesticide resistant strains and when new and challenging plant host are encountered. Our analyses will facilitate biochemical and toxicological studies of the role of *T*. *urticae* ABC transporters in spider mite physiology, and in particular the extraordinary host range and pesticide resistance development.

## Results and discussion

### Identification of spider mite ABC genes

**Figure 1:**
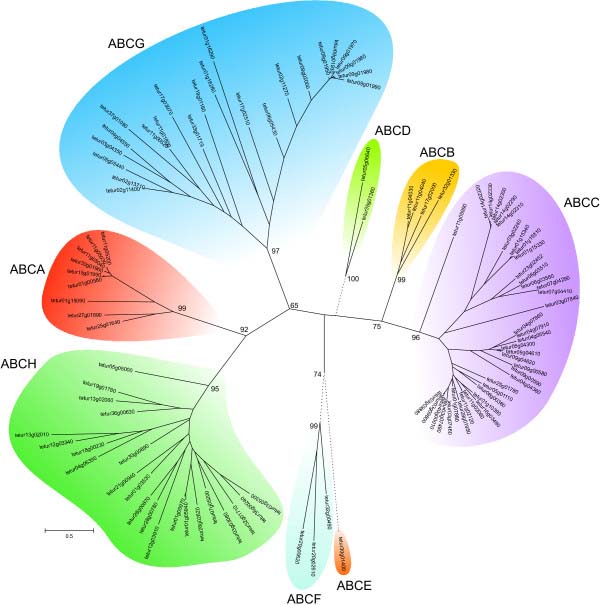
**Unrooted phylogenetic tree of N-terminal NBDs of 103 ABC proteins of *T*. *urticae.*** Amino acid sequences of NBDs were aligned using MUSCLE [122] and subjected to a maximum likelihood analysis using Treefinder [124]. For amino acid alignment, amino acid substitution model and likelihood score of the constructed phylogenetic tree see Additional file 9 and Additional file 10. Numbers at important nodes represent the bootstrap values resulting from 1000 pseudoreplicates (LR-ELW). The scale bar represents 0.5 amino-acid substitutions per site. The different ABC protein subfamilies are indicated by shaded colors. *T*. *urticae* ABC protein sequences can be found in Additional file 12.

We identified 103 putative ABC genes in the genome of *T*. *urticae* (Table 1). To our knowledge, this is the largest number of ABC genes reported for any metazoan species so far [8,9,13,45]. Of all organisms sequenced to date, only the protozoan ciliate *Tetrahymena thermophila* has more ABC genes [46]. A maximum likelihood phylogenetic analysis grouped the *T*. *urticae* ABC proteins into each of the eight known ABC subfamilies with high bootstrap support (Figure 1, Additional file 1). We identified 9, 4, 39, 2, 1, 3, 23 and 22 ABC proteins belonging to the ABCA, ABCB, ABCC, ABCD, ABCE, ABCF, ABCG and ABCH subfamilies, respectively (Table 2). Significant homology (E-value ≤ e^-4^) with one of the 103 putative *T*. *urticae* ABC genes was found at an additional 41 loci in the *T*. *urticae* genome. However, gene models at these loci, most of which had homology to the ABCC or ABCH subfamilies, lacked one or both vital domains (NBDs, TMD) of canonical ABC genes. These likely represent gene fragments or pseudogenized genes, and were excluded from detailed analysis (Additional file 2). From the 103 full-length *T*. *urticae* ABC genes, almost half (48) are located on only 5 genomic scaffolds (11, 9, 6, 11 and 10 ABC genes on scaffold 1, 3, 4, 9 and 11, respectively). In addition, a high complexity was observed within gene structures, with exon numbers ranging from 1 to 20 (Table 1). To determine the accurate evolutionary position of the 103 *T*. *urticae* ABC proteins, phylogenetic analyses, including full-length ABC protein sequences from the draft genomes of *T*. *urticae* and *D*. *pulex* and from the finished genomes of *C*. *elegans*, *D*. *melanogaster* and *H*. *sapiens*, were performed for each subfamily separately. The results of these analyses are discussed below.

**Table 1:** **Characterisation of 103 *T***. ***urticae *ABC proteins**

**Subfamily**	**Tetur ID**^**1**^	**Name**	**Length(AA)**	**Strand**	**Exons**	**Topology**^**2**^	**N**-**Glc**^**3**^	**O**-**Glc**^**4**^
A (9)	tetur01g00580	TuABCA-01	1659	-	5	(6TM-NBD)_2_	5	
	tetur01g15090	TuABCA-02	1639	+	4	(7/8TM-NBD)_2_	2	1
	tetur11g05030	TuABCA-03	1670	-	5	(5/6TM-NBD)_2_	3	
	tetur11g05040	TuABCA-04	1670	-	5	(6TM-NBD)_2_	2	
	tetur11g05200	TuABCA-05	1672	-	5	(6TM-NBD)_2_	2	
	tetur15g01990	TuABCA-06	1666	+	5	(6TM -NBD)_2_	3	
	tetur25g01640	TuABCA-07	2302	+	20	(6TM-NBD)_2_	8	
	tetur27g01890	TuABCA-08	2082	+	2	(7/4TM-NBD)_2_	5	27
	tetur30g01960	TuABCA-09	1651	-	5	(6/8TM-NBD)_2_	3	
B (4)	tetur11g04030	TuABCB-01	1294	+	15	(6TM-NBD)_2_	4	
	tetur11g04040	TuABCB-02	1292	+	16	(6TM-NBD)_2_	4	
	tetur17g02000	TuABCB-03	688	-	8	4TM-NBD	1	
	tetur32g01330	TuABCB-04	656	-	8	5TM-NBD	3	
C (39)	tetur01g07880	TuABCC-01	1507	+	11	5TM-(5TM-NBD)_2_	1	2
	tetur01g10390	TuABCC-02	1529	+	11	7TM-(5TM-NBD)_2_		
	tetur01g15310	TuABCC-03	1299	+	15	(5/6TM-NBD)_2_	5	1
	tetur01g15330	TuABCC-04	1328	+	14	(7TM-NBD)_2_	5	
	tetur01g15340	TuABCC-05	1338	+	14	(8/7TM-NBD)_2_	3	
	tetur03g02240	TuABCC-06	1375	-	12	(6TM-NBD)_2_	2	
	tetur03g07460	TuABCC-07	1481	-	11	6TM-(5TM-NBD)_2_	2	
	tetur03g07490	TuABCC-08	1483	-	11	6TM-(5TM-NBD)_2_	1	
	tetur03g07840	TuABCC-09	1457	-	5	5TM-(6TM-NBD)_2_	1	
	tetur03g09800	TuABCC-10	1490	+	11	6TM-(5TM-NBD)_2_		1
	tetur03g09880	TuABCC-11	1495	+	11	4TM-(5TM-NBD)_2_	1	1
	tetur04g04360	TuABCC-12	1522	+	5	8TM-(5TM-NBD)_2_	3	
	tetur04g05540	TuABCC-13	1503	+	8	6TM-(5TM-NBD)_2_	5	
	tetur04g07860	TuABCC-14	1501	-	8	7TM-(4TM-NBD)_2_	3	
	tetur04g07910	TuABCC-15	1493	-	8	6TM-(5TM-NBD)_2_	2	
	tetur05g01110	TuABCC-16	1522	-	11	4TM-(5TM-NBD)_2_	6	1
	tetur05g04300	TuABCC-17	1490	+	8	5TM-(6TM-NBD)_2_	6	2
	tetur06g00360	TuABCC-18	1516	+	11	6TM-(5TM-NBD)_2_	4	1
	tetur06g03510	TuABCC-19	1343	+	6	(6TM-NBD)_2_	2	
	tetur06g03560	TuABCC-20	1339	+	6	(5/7TM-NBD)_2_	2	
	tetur07g04290	TuABCC-21	1324	-	5	(6TM-NBD)_2_	1	
	tetur07g04410	TuABCC-22	1324	+	5	(6TM-NBD)_2_	1	
	tetur09g00580	TuABCC-23	1506	+	8	7TM-(6TM-NBD)_2_	2	
	tetur09g00590	TuABCC-24	1496	+	9	6TM-(6TM-NBD)_2_	1	
	tetur09g04610	TuABCC-25	1521	+	8	7TM-(6TM-NBD)_2_	4	1
	tetur09g04620	TuABCC-26	1501	+	8	6TM-(5TM-NBD)_2_	5	
	tetur11g02060	TuABCC-27	1499	-	11	4TM-(6TM-NBD)_2_	1	
	tetur11g02120	TuABCC-28	1492	+	11	6TM-(4TM-NBD)_2_	2	1
	tetur11g05990	TuABCC-29	1683	-	15	5TM-(6TM-NBD)_2_	6	
	tetur14g02290	TuABCC-30	1309	-	16	(5/7TM-NBD)_2_	4	
	tetur14g02300	TuABCC-31	1312	-	16	(5/7TM-NBD)_2_	5	
	tetur14g02310	TuABCC-32	1308	-	16	(6/7TM-NBD)_2_	3	1
	tetur14g02320	TuABCC-33	1309	-	16	(7TM-NBD)_2_	2	1
	tetur14g02330	TuABCC-34	1309	-	16	(5/7TM-NBD)_2_	2	
	tetur16g03480	TuABCC-35	1513	-	11	6TM-(5TM-NBD)_2_	2	1
	tetur23g02452	TuABCC-36	1344	-	6	(8/6TM-NBD)_2_	2	
	tetur25g01780	TuABCC-37	1525	+	12	5TM-(5TM-NBD)_2_		
	tetur28g01950	TuABCC-38	1287	+	7	(6TM-NBD)_2_	3	
	tetur40g00010	TuABCC-39	1490	+	11	5TM-(5TM-NBD)_2_		1
D (2)	tetur05g06640	TuABCD-01	829	+	4	5TM-NBD	2	1
	tetur35g01360	TuABCD-02	656	+	5	5TM-NBD	4	
E (1)	tetur30g01400	TuABCE-01	614	-	2	NBD-NBD		
F (3)	tetur20g02610	TuABCF-01	612	+	4	NBD-NBD	3	
	tetur29g00620	TuABCF-02	584	+	1	NBD-NBD	3	
	tetur32g00490	TuABCF-03	718	-	4	NBD-NBD	4	
G (23)	tetur01g16280	TuABCG-01	915	-	6	NBD-7TM	1	1
	tetur01g16290	TuABCG-02	643	+	3	NBD-6TM	1	
	tetur02g11270	TuABCG-03	689	+	11	NBD-6TM	1	
	tetur02g11400	TuABCG-04	811	-	2	NBD-6TM	3	1
	tetur02g13710	TuABCG-05	810	-	2	NBD-6TM	3	
	tetur03g04350	TuABCG-06	687	-	2	NBD-6TM	2	
	tetur04g04550	TuABCG-07	720	+	2	NBD-6TM	3	1
	tetur05g05440	TuABCG-08	721	+	2	NBD-8TM	1	1
	tetur06g05430	TuABCG-09	646	-	12	NBD-7TM	3	
	tetur09g01930	TuABCG-10	685	+	9	NBD-5TM	2	
	tetur09g01950	TuABCG-11	682	+	9	NBD-5TM	1	
	tetur09g01960	TuABCG-12	683	+	9	NBD-5TM	3	
	tetur09g01970	TuABCG-13	687	+	9	NBD-6TM	3	
	tetur09g01980	TuABCG-14	680	+	9	NBD-6TM	4	
	tetur09g01990	TuABCG-15	687	+	9	NBD-6TM	2	4
	tetur09g02000	TuABCG-16	682	+	9	NBD-7TM	3	
	tetur11g00520	TuABCG-17	792	-	1	NBD-8TM	2	3
	tetur11g01800	TuABCG-18	790	-	1	NBD-8TM	2	3
	tetur17g02510	TuABCG-19	832	-	4	NBD-4TM	2	1
	tetur17g03970	TuABCG-20	700	-	2	NBD-8TM	5	
	tetur19g01160	TuABCG-21	775	-	1	NBD-7TM	4	2
	tetur33g01719	TuABCG-22	760	+	1	NBD-6TM	1	
	tetur37g01090	TuABCG-23	779	-	1	NBD-7TM	1	14
H (22)	tetur01g03530	TuABCH-01	827	+	1	NBD-8TM	6	7
	tetur01g05940	TuABCH-02	766	-	6	NBD-6TM	2	2
	tetur01g05970	TuABCH-03	734	-	6	NBD-6TM	1	1
	tetur03g03080	TuABCH-04	767	-	2	NBD-8TM	3	
	tetur03g05300	TuABCH-05	738	-	1	NBD-6TM	1	
	tetur04g06390	TuABCH-06	707	+	1	NBD-7TM	2	1
	tetur05g05000	TuABCH-07	713	+	1	NBD-7TM	3	
	tetur07g05200	TuABCH-08	764	-	4	NBD-6TM		
	tetur12g03340	TuABCH-09	744	-	1	NBD-5TM	1	
	tetur12g03910	TuABCH-10	713	+	1	NBD-7TM	3	
	tetur13g02010	TuABCH-11	703	-	1	NBD-8TM	2	1
	tetur13g02060	TuABCH-12	723	-	1	NBD-7TM	3	
	tetur18g00230	TuABCH-13	735	-	2	NBD-8TM	4	20
	tetur19g01780	TuABCH-14	692	-	1	NBD-7TM	5	2
	tetur21g00940	TuABCH-15	716	+	2	NBD-6TM	1	1
	tetur26g02620	TuABCH-16	708	-	10	NBD-7TM	2	4
	tetur28g00780	TuABCH-17	726	-	1	NBD-8TM	1	2
	tetur28g00870	TuABCH-18	720	+	1	NBD-7TM	3	1
	tetur30g00890	TuABCH-19	721	-	1	NBD-7TM	2	
	tetur32g01710	TuABCH-20	804	-	1	NBD-6TM	2	4
	tetur36g00240	TuABCH-21	777	-	1	NBD-6TM	3	1
	tetur36g00630	TuABCH-22	704	-	1	NBD-6TM	1	

^1^*T*. *urticae* gene models can be accessed at the ORCAE genome portal, http://bioinformatics.psb.ugent.be/orcae/overview/Tetur[120].

^2^ transmembrane helices (TMs) were predicted using the SCAMPI server [128] while nucleotide binding domains (NBD) were determined using the ScanProSite facility [121] and Prosite profile PS50893; ( )_2_ indicates that the *T*. *urticae* ABC protein is a full transporter.

^3^ N-glycosylation sites were predicted using NetNGlyc 1.0 server [131]; only N-glycosylation site with a “potential” score > 0.5 and with a jury agreement were taken into account.

^4^O-glycosylation sites were predicted using NetOGlyc 3.1 server [132]; if the G-score was higher than 0.5 the residue was considered to be O-glycosylated; in this table the total number of O-glycosylated sites (glycosylated serines and threonines) is shown.

**Table 2:** **ABC subfamilies in *S***. ***cerevisiae***, ***C***. ***elegans***, ***H***. ***sapiens***, ***D***. ***melanogaster***, ***T***. ***castaneum***, ***D***. ***pulex *and *T***. ***urticae***

ABC subfamily*	***S. cerevisiae***	***C. elegans***	***H. sapiens***	***D. melanogaster***	***T. castaneum***	***D. pulex***	***T. urticae***
A	0	7	12	10	9	4	10
B-full	1	14	4	4	4	2	2
B-half	3	10	7	4	2	5	2
C	6	9	12	14	35	7	39
D	2	5	4	2	2	3	2
E	2	1	1	1	1	1	1
F	6	3	3	3	3	4	3
G	10	9	5	15	13	24	23
H	0	0	0	3	3	15	22
**TOTAL**	30	58	48	56	73	65	**103**

* numbers were derived from [8,9,11] and this study; one additional ABCG transporter was identified for *D*. *pulex* (Dappu1_300887) in this study resulting in 65 *D*. *pulex* ABCs; a *C*. *elegans* ABCB half transporter discussed by [62] was added to the number of *C*. *elegans* ABCB half transporters resulting in 58 ABC transporters for *C*. *elegans.*

**The ABCA family** comprises 9 full transporters (hereafter FTs) in *T*. *urticae*. In contrast to plants and some insects, no ABCA half transporters (hereafter HTs) were identified (Table 1) [9,12,47]. The *T*. *urticae* ABCA subfamily contains the largest *T*. *urticae* ABC protein, tetur25g01640 (2302 amino acids) (Table 1). ABCAs share a distinct set of characteristics across species: an extracellular loop between the first and second transmembrane helices (TMs), a conserved motif downstream of each NBD [48] and a conserved motif at the N-terminus (xLxxKN, [49]). All *T*. *urticae* ABCAs have a characteristic extracellular loop between the first and the second TMs of each TMD (see Additional file 3 for position of TMs). The conserved motif downstream of each NBD was, except for tetur25g01640, present in all *T*. *urticae* ABCAs, while the N-terminus conserved motif could only be found in a single *T*. *urticae* ABCA (tetur25g01640). Instead of xLxxKN, the remainder of *T*. *urticae* ABCAs harbor either a xMxxKD/S (7) or xLxxHR (1) N-terminal motif. A phylogenetic analysis of metazoan ABCAs is shown in Additional file 4. Six *T*. *urticae* ABCAs (tetur01g00580, tetur11g05030, tetur11g05040, tetur11g05200, tetur15g01990 and tetur30g01960) clustered together with high bootstrap support. These six ABCAs show high amino acid identity (62.3-94.1%, Additional file 5) and have identical exonic structure (Additional file 3), indicating they might have arisen by recent duplication events. Together with tetur01g15090, they form a sister-group with *D*. *melanogaster* CG31731, an ABCA reported to be down regulated in the salivary glands of an *E93* mutant of *D*. *melanogaster* ([50]; the early ecdysone responsive gene *E93* is a critical regulator of programmed cell death during *D*. *melanogaster* metamorphosis). *D*. *melanogaster* CG31731 and the seven *T*. *urticae* ABCA genes cluster together, albeit with moderate bootstrap support, with a group of *C*. *elegans* ABCA transporters. The latter contains Ced-7, which is involved in the engulfment of cell corpses during programmed cell death in *C*. *elegans*[51].

Further, tetur27g01890 and *D*. *melanogaster* CG34120 form a sister clade of human ABCA12 and ABCA13, while tetur25g01640, *D*. *pulex* Dappu1-312055 and Dappu1-312056 cluster with human ABCA1, ABCA2, ABCA4 and ABCA7 (Additional file 4). These human ABCAs contain conserved predicted N-glycosylation sites at N400, N1453 and N1637 of human ABCA1 [48]. In addition, it has been experimentally shown that *D*. *melanogaster* CG34120 is also glycosylated at an asparagine (N272, at NASFEEL motif of CG34120 [52]) aligning with one of these conserved sites (N400 of human ABCA1). The tetur27g01890 and tetur25g01640 proteins also have many predicted N-glycosylation sites (Table 1) of which at least one (N303 in tetur27g01890; N1705 in tetur25g01640) is shared with those conserved in human ABCA1, 2, 4, 7 and 12. In humans, these ABCAs have highly specialized roles in phospho- and sphingolipid export [48]. For example, human ABCA1 controls the initial steps leading to high-density lipoprotein (HDL) formation at the cell membrane and is crucial for reverse cholesterol transport from peripheral tissues to the liver [53]. Human ABCA12 works as an epidermal keratinocyte lipid transporter and a defective ABCA12 results in loss of the skin lipid barrier [54,55]. Although we cannot assign such highly specific roles to the two *T*. *urticae* ABCA orthologues above, they may also be involved in lipid transport processes.

**Figure 2:**
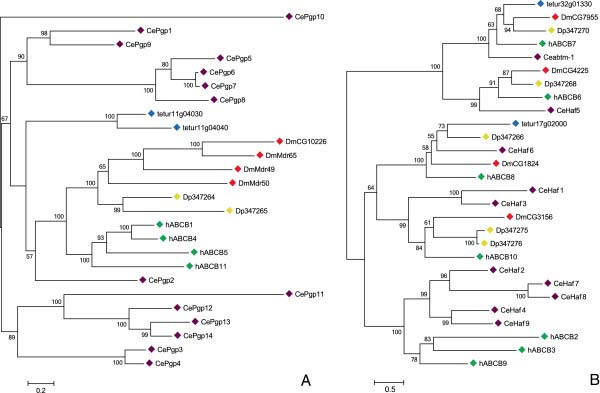
**Phylogenetic analysis of ABCB full and half transporters of five metazoan species: (A) ABCB full transporters, (B) ABCB half transporters.** Full-length ABC proteins were aligned using MUSCLE [122] and subjected to a maximum likelihood analysis using Treefinder [124]. The resulting tree was midpoint rooted. For amino acid alignment, amino acid substitution model and likelihood score of the constructed phylogenetic tree see Additional file 9 and Additional file 10. Numbers at the branch point of each node represent the bootstrap value resulting from 1000 pseudoreplicates (LR-ELW). Species, abbreviations, and color codes are: Ce, *C*. *elegans* (purple); h, *H*. *sapiens* (green); Dm, *D*. *melanogaster* (red); Dp, *D*. *pulex* (yellow); and tetur, *T*. *urticae* (blue). The scale bar represents 0.2 and 0.5 amino-acid substitutions per site in Figure 2A and Figure 2B, respectively. Accession numbers of metazoan ABC protein sequences can be found in Additional file 11 while *T*. *urticae* ABC protein sequences can be found in Additional file 12.

**The ABCB subfamily** consists of 2 FTs and 2 HTs in *T*. *urticae* (Table 1). A phylogenetic analysis of ABCB FTs revealed that transporters of each species in the analysis clustered into separate clades, confirming an earlier hypothesis by Sturm et al. that this subfamily has diversified through lineage-specific duplications [11] (Figure 2A). This diversification hypothesis is supported in mites by the fact that the *T*. *urticae* ABCB FTs, tetur11g04030 and tetur11g04040, have well-supported phylogenetic clustering, similar exon patterns (15 and 16 exons for *tetur11g04030* and *tetur11g04040*, respectively (see Additional file 3)) and high amino acid identity (64.8%, see Additional file 5). *T*. *urticae* ABCB FTs form a sistergroup to a clade of *C*. *elegans*, *H*. *sapiens* and *D*. *melanogaster* ABCB FTs. The function of most members of this clade has been well documented in literature. Human ABCB FT, originally termed P-glycoproteins (P-gps) but now also known as multiple drug resistance (MDR) proteins, are among the best characterized ABC pumps and have been shown to be involved in transport of hydrophobic substrates including drugs, lipids, steroids, xenobiotics and peptides (Dean et al. 2001). The precise role of their orthologues in *Drosophila* has been a focus of recent study. *D*. *melanogaster* Mdr65 has been shown to function as an orthologue of human ABCB1/MDR1, a major ABC transporter of cytotoxic xenobiotics at the human blood–brain barrier, and is required for chemical protection of the fruitfly brain [56] while Mdr49 has been shown to be essential in germ cell migration [21]. Interestingly, arthropod ABCB FT orthologues have frequently been linked to pesticide resistance [14,26]. For example, inhibition of a *H*. *virescens* orthologue of human ABCB1 by the P-gp inhibitor quinidine decreased the toxicity of thiodicarb by 12.5-fold in a resistant strain, compared to 1.8-fold in a susceptible strain [28]. Recently, it was found that pretreatment of *D*. *melanogaster* with the P-gp inhibitor verapamil reduced the toxicity of DDT by 10-fold in a resistant strain [57]. The involvement of P-gps in pesticide resistance is probably best documented for ivermectin resistance. This compound has been shown to be a substrate for both mammalian as insect-pgps and several cases of P-gp associated ivermectin resistance have been reported [26,58-60].

A phylogenetic analysis of ABCB HTs revealed, as was also shown by Sturm et al. [11], clear orthologous relationships between ABCB HTs, suggesting they have evolutionary conserved roles in metazoan species (Figure 2B). In the case of *T*. *urticae* ABCB HTs, an orthologous relationship between tetur32g01330 and *D*. *melanogaster* CG7955, *D*. *pulex* Dappu1-347270, *C*. *elegans* ABTM-1 and human ABCB7 was found, while tetur17g02000 groups together with *D*. *melanogaster* CG1824, *D*. *pulex* Dappu1-347266, *C*. *elegans* Haf-6, and human ABCB8. As both tetur32g01330 and tetur17g02000 are predicted (data not shown) to have a mitochondrial targeting signal, these *T*. *urticae* transporters are most likely trafficked to the mitochondria, as has been demonstrated for their human orthologues (human ABCB7 and ABCB8) [61]. This suggests that tetur32g01330 and tetur17g02000 fulfill a similar role as their human orthologues. The human ABCB7 protein plays a crucial role in iron homeostasis in the cytoplasm and mutations in this gene have been linked to several diseases [61]. Recently, it was also shown that disruption of the *C*. *elegans* orthologue (ABTM-1) of human ABCB7 induced oxidative stress and premature cell death [62]. Furthermore, an orthologue (GenBank acc. No. AAEL006717) of human ABCB7 in the dengue vector, *Aedes aegypti*, was reported to be upregulated in an insecticide resistant strain [9,63]. The function of human ABCB8 is not well understood, but it was shown to mediate resistance against the chemotherapeutic agent doxorubicin in melanoma cells [64,65]. Also, Ichikawa et al. found that disruption of the mouse orthologue of human ABCB8 lead to cardiomyopathy and decreased mitochondrial iron export [66]. Orthologues of the remaining human mitochondrial transporters, ABCB6 and ABCB10, could be identified in *D*. *pulex*, *D*. *melanogaster* and *C*. *elegans* but were not found in *T*. *urticae*[1,11] (Figure 2B). Interestingly, the localization of human ABCB6 is currently under debate, as some studies suggest that it is located in lysosomes [67]. Finally, similar to Sturm et al. [11], we did not identify arthropod orthologues of human ABCB HTs related to antigen processing (human ABCB2, ABCB3 and ABCB9) (Figure 2B).

**The ABCC subfamily** consists of 39 transporters in *T*. *urticae*. To our knowledge, this is the largest number of ABCC transporters reported in any metazoan species, including the flour beetle, *T*. *castaneum*, which also has an exceptionally large number of ABCCs [8,9] (Table 2). ABCC proteins are FTs, bearing 2 TMDs and 2 NBDs, with diverse functions: ion transport, cell-surface receptor activity and translocation of a broad array of substrates like drugs, cyclic nucleotides, endogenous compounds and their glutathione conjugates and glutathione [19,68-70]. Because of their ability to extrude drugs, many of these ABCCs are also termed multidrug resistance associated proteins (MRPs) [1]. MRPs can be categorized according to the presence or absence of a third N-terminal transmembrane-spanning domain (TMD_0_) . In humans, “long” MRPs like ABCC1, 2, 3, 6 and 10 (MRP1, 2, 3, 6 and 7, respectively) have such a TMD_0_ while “short” human MRPs like ABCC4, 5, 11 and 12 do not (MRP4, 5, 8 and 9, respectively) [71]. In addition to MRPs, the ABCC family also harbors the cystic fibrosis transmembrane conductance regulator (CFTR, human ABCC7) and sulfonylurea receptors (human ABCC8/SUR1 and ABCC9/SUR2) [1].

**Figure 3:**
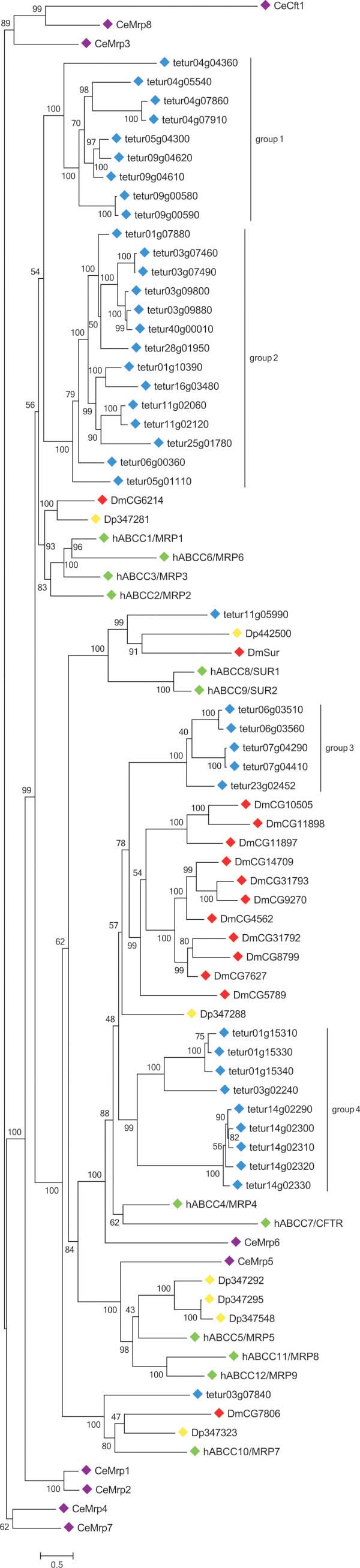
Phylogenetic analysis of ABCC proteins of five metazoan species. See the legend of Figure 2 for procedure and display details.

In our phylogenetic analysis, 23 *T*. *urticae* ABCCs clustered with *D*. *melanogaster* CG6214, *D*. *pulex* Dappu1-347281 and a group of human “long” MRPs (MRP1, 2, 3 and 6) (Figure 3). Twenty-two of the transporters (size ranging from 1481 to 1529 amino acids) from this *T*. *urticae* ABCC clade also have a TMD_0_, while we could not identify this additional transmembrane domain in tetur28g01950 (1287 amino acids) (Table 1), Additional file 3). Within this *T*. *urticae* ABCC clade, transporters clustered into 2 distinct groups. One group (“group 1”) consists of 8 *T*. *urticae* ABCC genes, each with 7 introns, and *tetur04g04360*, which has only 4 introns (see Additional file 3). Interestingly, tetur04g04360 is positioned at the basal node, indicating that intron gain events may have occurred in this group (Figure 3). The other group (“group 2”) consists of 14 *T*. *urticae* ABCC genes: 12 with 10 introns, *tetur25g01780* with 11 introns and *tetur28g01950* having only 6 introns (Additional file 3). The intron loss in *tetur28g01950* can be directly linked to the lack of the TMD_0_ (see above, Additional file 3), while the nature of the intron gain event in *tetur25g01780* is unclear (the extra intron contains stop codons in each frame, and is located in a non conserved region). Human MRP1, MRP2, MRP3 and MRP6 along with *D*. *melanogaster* CG6214 and *D*. *pulex* Dappu1-347281 form a sister clade of *T*. *urticae* ABCC groups 1 and 2 (Figure 3). These proteins have been extensively studied as transporters of natural product drugs like anthracyclines and plant alkaloids [72]. MRPs also share many substrates with human P-gps (see above) but, while P-gps transport drugs in their original form, MRPs mostly transport their glucuronate, sulfate and glutathione (GSH) conjugates. In the latter case, GSH is fused by GSTs with xenobiotics or their metabolites and finally transported out of the cell by MRPs [46,72,73]. In humans, GSTs from the alpha, mu and pi-class have been reported to act in synergy with MRPs [74]. Intriguingly, a clear expansion of GSTs in the *T*. *urticae* genome was found for the delta and mu GST subclasses, the latter of which is not present in insects and until recently was believed to be vertebrate specific [37,75]. Future studies should point out if there is a coordinated action between the GSTs of these subfamilies and the many MRP orthologues of *T*. *urticae*.

Most of the biochemical properties of human MRP1 have been confirmed in its *D*. *melanogaster* orthologue CG6214 [76,77]. Recently, it was also found that exposure to the P450 mono-oxygenase inhibitor piperonylbutoxide and the antimetabolite/antifolate drug methotrexate alters the expression (27-fold and 1100-fold upregulation, respectively) of the *D*. *melanogaster* CG6214 gene in the Malpighian tubuli, which are organs known to play important roles in excretion and xenobiotic detoxification [78,79]. The orthologue (*PhABCC4*) of CG6214 in the human body louse, *Pediculus humanus*, has also been reported to be upregulated after exposure to the pesticide ivermectin. Moreover, injection of *PhABCC4* dsRNA in *P*. *humanus* female lice increased their sensitivity to ivermectin by 20-30% [60].

Fourteen spider mite ABCCs clustered with high bootstrap support in a group with 11 *D*. *melanogaster* ABCC proteins, human ABCC4/MRP4 and ABCC7 and *D*. *pulex* Dappu1-347288 (Figure 3). All fourteen *T*. *urticae* ABCCs show the structural properties of “short” MRPs (see above, Table 1), Additional file 3). Five of these *T*. *urticae* ABCCs group as a sister clade (“group 3”) of the 11 *D*. *melanogaster* ABCC proteins (Figure 3). Detailed physiological roles for most of the 11 *D*. *melanogaster* ABCC proteins are unknown. *D*. *melanogaster* CG10505 is regulated by heavy metals through the metal-responsive transcription factor 1 (MTF-1) and contributes to metal homeostasis [80]. *D*. *melanogaster* CG14709 controls responsiveness to O_2_ deprivation and might also be involved in oxidative stress response [81,82] while 31% of embryos of *D*. *melanogaster* CG7627 mutants were unable to heal wounds 16 h postwounding [83]. Recently, it was also shown that *D*. *melanogaster* CG4562, which is highly expressed in the midgut, was upregulated in *Cyp6g1* knockdown flies, indicating molecular crosstalk within a detoxification network [84]. Furthermore, a point mutation in lepidopteran homologs of this *D*. *melanogaster* ABCC group has been clearly linked with resistance against the *B*. *thuringiensis* Cry1A toxin [27,29]. Another 9 *T*. *urticae* ABCCs (“group 4”) form, together with *D*. *pulex* Dappu1-347288 and the 11 *D*. *melanogaster* and five *T*. *urticae* ABCCs (“group 3”, see above) a sister clade of human ABCC4 and ABCC7 (Figure 3). The function of *D*. *pulex* Dappu1-347288 is not known, but human ABCC7/CFTR acts as a chloride channel, a unique function not found in any other ABC transporter [69]. In our analysis, CFTR clustered with human ABCC4/MRP4, which is its closest ABC paralog according to Jordan et al. [69]. Human MRP4 (and MRP5) have the ability to transport a range of endogenous molecules involved in cellular signaling, like cyclic nucleotides, eicosanoids and conjugated steroid hormones [19]. As a drug transporter, MRP4 also stands out for its broad substrate specificity, covering antiviral, antibiotic, cardiovascular and cytotoxic agents [85].

Interestingly, many transporter genes in *T*. *urticae* ABCC groups 1–4 form closely related sister groups and show high amino acid identity between their corresponding protein sequences (see Additional file 5). Together with their conserved exon pattern (see Additional file 3), this strongly suggests that multiple tandem duplications underlie the proliferation of these genes. This is in contrast to the crustacean *D*. *pulex*, which has only few ABCC genes [11], and more closely resembles what is generally observed for insects, where gene duplication of ABCC genes occurs frequently [8,9,12].

Furthermore, clear orthologous relationships were found for the two remaining *T*. *urticae* ABCCs: tetur03g07840 and tetur11g05990. Tetur03g07840 clustered as an orthologue of *D*. *melanogaster* CG7806, *D*. *pulex* Dappu1-347323 and human ABCC10/MRP7 (Figure 3). Similar to its orthologues, tetur03g07840 has a TMD_0_[11,86] (Table 1), Additional file 3). The functions of *D*. *melanogaster* CG7806 and *D*. *pulex* Dappu1-34723 are not known. A growing understanding of the physiological role of human ABCC10/MRP7 is, on the other hand, beginning to emerge. Human MRP7 is distinct from other human ABCCs in that it shows little or no activity towards glutathione, sulfate conjugates and cyclic nucleotides, substrates that can be handled by other human MRPs (see above). Instead, human MRP7 is able to confer resistance to taxanes (which are diterpenes originally derived from plants of the genus *Taxus* and widely used as chemotherapy agents (e.g. docetaxel)) [86]. However, the presence of one-to-one orthologues in other metazoans, might indicate a more conserved function of this ABCC protein in this group of species.

Tetur11g05990 is located in the same clade as *D*. *pulex*, human and *D*. *melanogaster* sulfonylurea receptors (SURs). In contrast to vertebrates, the N-terminal SUR Interpro-motif (IPR000388) is not present in tetur11g05990 and other arthropod SURs (see OrthoDb group EOG531ZCW, [87]). However, the presence of a TMD_0_ typical for SURs and “long” MRPs [71], and the well-supported clustering with human ABCC8/SUR1 and ABCC9/SUR2 support the idea that tetur11g05990 is a SUR homologue. Four SUR subunits assemble into an octameric complex with four pore-forming subunits, characteristic for inwardly rectifying potassium (K_ir_) channels, to form ATP-sensitive potassium (K_ATP_) channels [68]. Three orthologues of these pore- forming subunits were also found in the *T*. *urticae* genome (tetur17g01380, tetur24g01270 and tetur24g01280 having a BLASTx E-value of 1e^-99^, 4e^-103^ and 3e^-103^ with *Ir* (*CG44159*) of *D*. *melanogaster*), suggesting that a functional K_ATP_ channel can be formed in *T*. *urticae*. K_ATP_ channels are involved in multiple physiological processes, with roles in glucose homeostasis, ischemic protection and innate immunity [68,88]. Intriguingly, in 2004 it was suggested that the SUR was the direct target of benzoylureas, a group of chitin synthesis inhibitors [89]. This was largely based on similar effects of glibenclamide, a well-known SUR inhibitor in humans and anti-diabetic drug, on the inhibition of chitin synthesis. However, it was later shown by Gangishetti et al. [90] that SUR is not expressed in the *D*. *melanogaster* epidermis, where chitin disruption is observed. Recently, based on genetic mapping of etoxazole resistance genes, it was suggested that the action of chitin synthesis inhibitors is mediated by a direct interaction with chitin synthase, a processive glycosyl transferase [43]. The lack of a role for SUR in chitin production, transport or metabolism is further confirmed by recent studies, where it was shown that the SUR receptor is dispensable for chitin synthesis in *D*. *melanogaster*[91], and RNAi knockdown of its orthologue in *T*. *castaneum* did not result into a phenotype [8]. Elucidating the role of SUR in *T*. *urticae* will therefore require additional studies.

Finally, no orthologues of human ABCC5, 11 and 12 were identified in *T*. *urticae*, although three orthologues were found in the genome of *D*. *pulex* (Figure 3), confirming earlier findings by Sturm et al. [11]. Surprisingly, a single nucleotide polymorphism in human ABCC11 was identified as the determinant of the human earwax type [92]. However, the potential roles of related transporters in other organisms (such as *D*. *pulex*) are not clear.

**The ABCD subfamily** harbors HTs that in humans are located in the peroxisome where they are involved in the import of long and branched chain acyl-coA into this organelle [93]. The *T*. *urticae* genome has 2 ABCD genes, *tetur05g06640* and *tetur35g01360* (Table 1). This number of ABCD genes equals those found in insects [9] while 3, 4 and 5 are found in the genomes of *D*. *pulex*, *H*. *sapiens* and *C*. *elegans*, respectively (Table 1). *T*. *urticae* ABCDs carry the EAA-like motif between TM4 and TM5 and the loop1 motif (L105, R108 and T109 (*S*. *cerevisiae* numbering)), both considered to be essential for canonical ABCD function [94]. The clear orthologous relationships we identified between *T*. *urticae* ABCDs and other metazoan ABCDs (Additional file 6) suggests that the function of *T*. *urticae* ABCDs is likely to be conserved with those in other metazoans.

**The ABCE and F** proteins are characterized by two linked NBDs, but lack TMDs and thus are involved in biological processes other than transport. The ABCE protein is essential in all eukaryotes examined to date and is one of the most conserved proteins known [95]. Human ABCE1 was first discovered as an inhibitor of RNase L [96], but was later found to have a more fundamental role in ribosome biogenesis and translation regulation [95]. In line with all eukaryotes to date, we found one ABCE protein (tetur30g01400) in *T*. *urticae* (Table 1) and Table 2) that has high amino acid identity (77.1%) with *D*. *melanogaster* ABCE1 (pixie) (Additional file 5). Similar to human ABCE, ABCF1 is involved in translation regulation, but probably does not play a role in ribosome biogenesis [97]. In most eukaryotes 3 ABCF genes are found, and *T*. *urticae* conforms to this expectation (Liu et al. [9], Table 1) and Table 2). The essential role of ABCE and ABCF genes was recently shown in the flour beetle, *T*. *castaneum*, where RNAi–mediated knockdown of members of the ABCE and F families resulted in 100% mortality in penultimate larvae [8].

A phylogenetic analysis of ABCE and ABCF proteins was performed together (Additional file 7). Tetur30g01400 grouped with metazoan ABCE1 orthologues, while each *T*. *urticae* ABCF (tetur20g02610, tetur29g00620 and tetur32g00940) clustered into well-supported separate clades with its metazoan orthologues, *C*. *elegans* F42A10.1 excluded (Additional file 7). The ABCE and ABCF subfamilies are highly conserved, and *T*. *urticae* ABCE1 and ABCFs probably have analogous roles as their orthologues in other metazoans.

**Figure 4:**
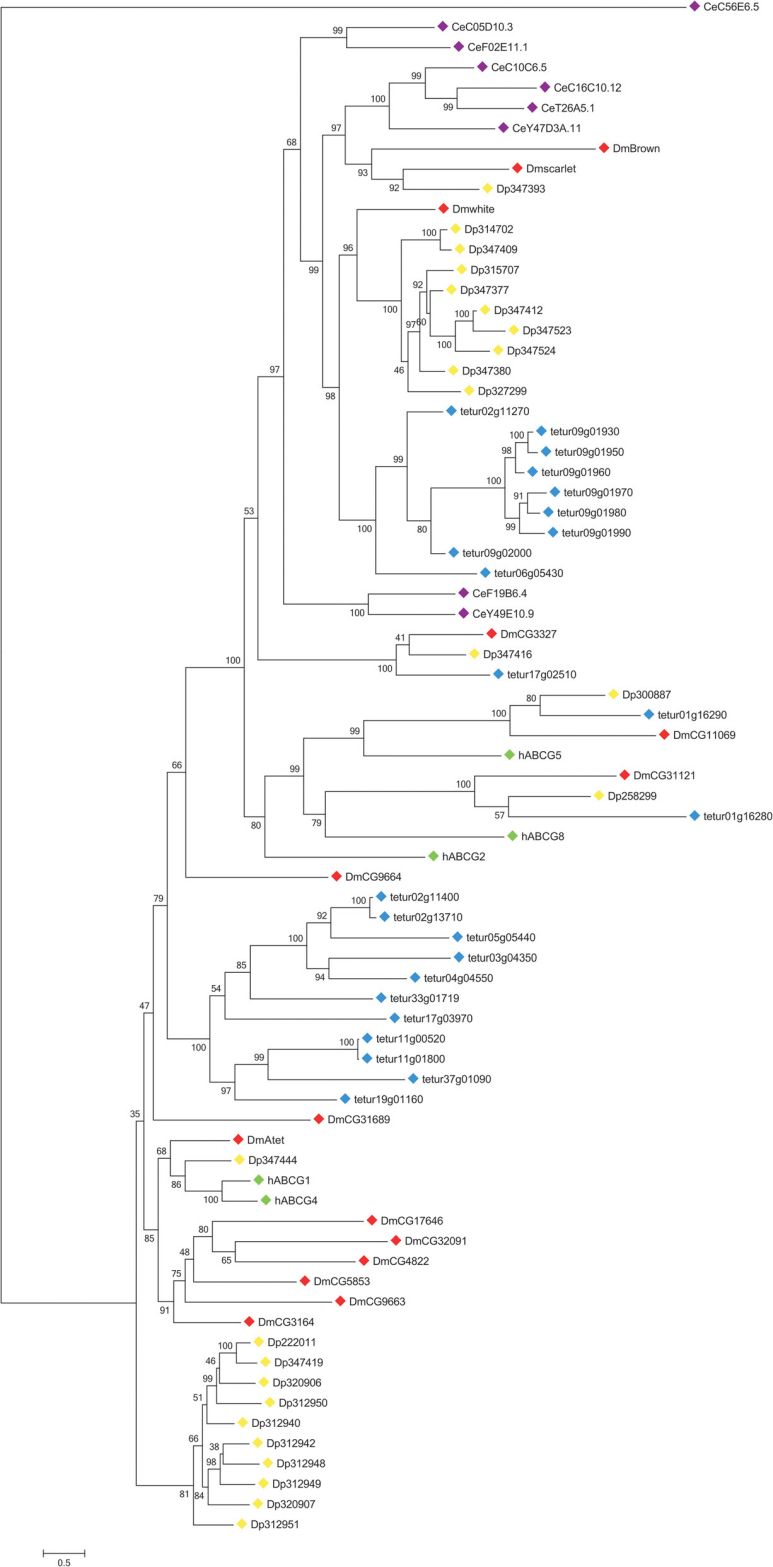
Phylogenetic analysis of ABCG proteins of five metazoan species. See the legend of Figure 2 for procedure and display details.

**The ABCG** transporter family is present in most metazoan species, fungi and plants such as *Arabidopsis*. For metazoan species, only ABCG HTs have been reported to date, while in plants and fungi also ABCG FTs are present [1,15,47]. In humans, ABCG HTs are primarily implicated in transport of endogenous and dietary lipids, while the human ABCG2 functions as a multidrug efflux pump [18,98]. Within the *T*. *urticae* genome we identified 23 ABCGs, all having a typical reverse domain organization (the NBD is localized to the N-terminal side of the TMD) (Table 1), see Additional file 3). A similar number of ABCGs has also been found in *D*. *pulex*, and is the highest reported among metazoan species [11] (Table 2). According to Sturm et al. [11], the high number of ABCG genes in *D*. *pulex* and *D*. *melanogaster* genomes is due to extensive lineage specific duplications. Our phylogenetic analysis confirms this hypothesis not only for these two arthropod species but also for *T*. *urticae*, with twenty out of 23 ABCGs grouping into one of the two *T*. *urticae* specific clades (Figure 4).

One clade (bottom of tree, Figure 4) consists of 11 *T*. *urticae* ABCGs, each having a maximum of 1 intron (see Additional file 3). Another *T*. *urticae* specific ABCG clade comprises 9 transporters of which 7 are located next to each other on scaffold 9. These seven ABCGs show high amino acid identity (between 50.8 and 91.7%) and have a conserved exon pattern (9 exons), indicating a common origin by successive tandem duplication events. Together with tetur06g05430 and tetur02g11270 they form a well-supported sister clade of *D*. *melanogaster* white and its *D*. *pulex* orthologues. Interestingly, no orthologues of *D*. *melanogaster* ABCGs brown and scarlet were found in *T*. *urticae*, while only one *D*. *pulex* orthologue of scarlet could be identified (Figure 4, [11]). About a century ago, the discovery of *D*. *melanogaster white* mutants with a remarkable eye-color phenotype marked the beginning of *Drosophila* genetics. As a consequence, *D*. *melanogaster white* is one of the most intensively studied fruit fly genes [99]. *D. melanogaster* white dimerises with either *D*. *melanogaster* scarlet or brown to form a transporter involved in the uptake of pigment precursors (guanine and tryptophan) in cells of developing compound and simple eyes [22]. *T*. *urticae* has, in contrast to *D*. *pulex* and *D*. *melanogaster*, no compound eyes and only four simple eyes (ocelli) [100]. Although no *T*. *urticae* orthologues of scarlet or brown were identified, dimerisation between the nine *T*. *urticae* co-orthologues of *D*. *melanogaster* white might result in a transporter capable of translocating pigment precursors into the cells of the spider mite ocelli (which have red pigment, as in *D*. *melanogaster*). However, these transporters might also have other functions besides transporting pigment precursors, as in other species roles have been documented in courtship behavior [101,102], transport of biogenic amines [103] and uptake of uric acid [104] as was shown for *D*. *melanogaster* white and/or its *B*. *mori* orthologue.

In the middle of the ABCG phylogenetic tree, tetur01g16280 clustered with human ABCG8, *D*. *melanogaster* CG31121 and *D*. *pulex* Dappu1-258299, while tetur01g16290 clustered with human ABCG5, *D*. *melanogaster* CG11069, and *D*. *pulex* Dappu1-300887. *C*. *elegans* orthologues of human ABCG5/8 could not be identified (Figure 4). Similar to human *ABCG5*/*8*, *D*. *melanogaster CG31121*/*CG11069*[105] and *D*. *pulex Dappu1*-*258299*/*Dappu1*-*300887*[106], *tetur01g16280* and *tetur01g16290* are found juxtaposed in a head to head orientation. Annilo et al. [7] have suggested an evolutionary constraint on the separation of these genes, probably for the maintenance of shared regulatory regions. In humans, ABCG5 and ABCG8 are both glycoproteins and obligate heterodimers that limit intestinal absorption and promote biliary excretion of neutral sterols [107]. Both tetur01g16280 and tetur01g16290 have at least one well-predicted glycosylation site (Table 1). Together with their head-to-head arrangement and the well-supported clustering with human ABCG8 and 5, it seems likely that these *T*. *urticae* ABCGs have similar functions as their human counterparts.

A clear orthologous relationship was found between tetur17g02510, *D*. *melanogaster* CG3327 and *D*. *pulex* Dappu1-347416. *D*. *melanogaster* CG3327, also known as E23 (Early gene at 23), is a 20-OH ecdysone (20E) induced ABC transporter that is capable of regulating 20E responses during metamorphosis, probably by removing 20E from cells [108]. Recently, Broehan et al. [8] showed through RNAi-mediated knockdown experiments and expression profiling that the *T*. *castaneum* orthologue of E23 (TcABCG-8A) appears to serve a similar function in metamorphosis. In addition, it is also believed that E23 controls the circadian clock in adult flies through ecdysone-mediated expression of the clock gene *vrille*[109]. Interestingly, it was shown that not only the *B*. *mori* orthologue of *E23* (BmABC010557) but also four other midgut-specific *B*. *mori* ABCG genes (*BmABC005226*, *BmABC005203*, *BmABC005202* and *BmABC010555*) could be induced by 20E [9]. As *T*. *urticae* uses a different molting hormone (ponasterone A instead of 20E) compared to arthropods [37], future experiments are required to establish if tetur17g02510 has a similar function as its insect counterparts, and more specifically whether it can be induced by ponasterone A.

*T*. *urticae* orthologues of human ABCG1 and 4 were not identified in the phylogenetic analysis of ABCG transporters, and only one was found in *D*. *melanogaster* (Atet) and *D*. *pulex* (Dappu1-34744) (Figure 4). The function of human ABCG4 is not well understood, while it is proposed that human ABCG1 functions in conjunction with human ABCA1 and is involved in cholesterol homeostasis [18,98]. The *D*. *melanogaster* orthologue of human ABCG1 (Atet) has been poorly characterized, but is expressed in the trachea [110]. Finally, no clear orthologues of human ABCG2 were identified (Figure 4). This transporter is the most thoroughly characterized human ABCG and is capable of transporting an array of substrates, including anticancer drugs [18,19,98]. Because of this feature, it has been proposed that arthropod ABCGs could be involved in pesticide resistance [14]. However, to the best of our knowledge there has only been two studies that correlated increased arthropod ABCG expression levels with resistance. In both reports, however, no functional evidence was obtained [13,111]. In Fungi on the other hand, several cases of ABCG FTs involved in fungicide resistance, have been reported [112].

**Figure 5:**
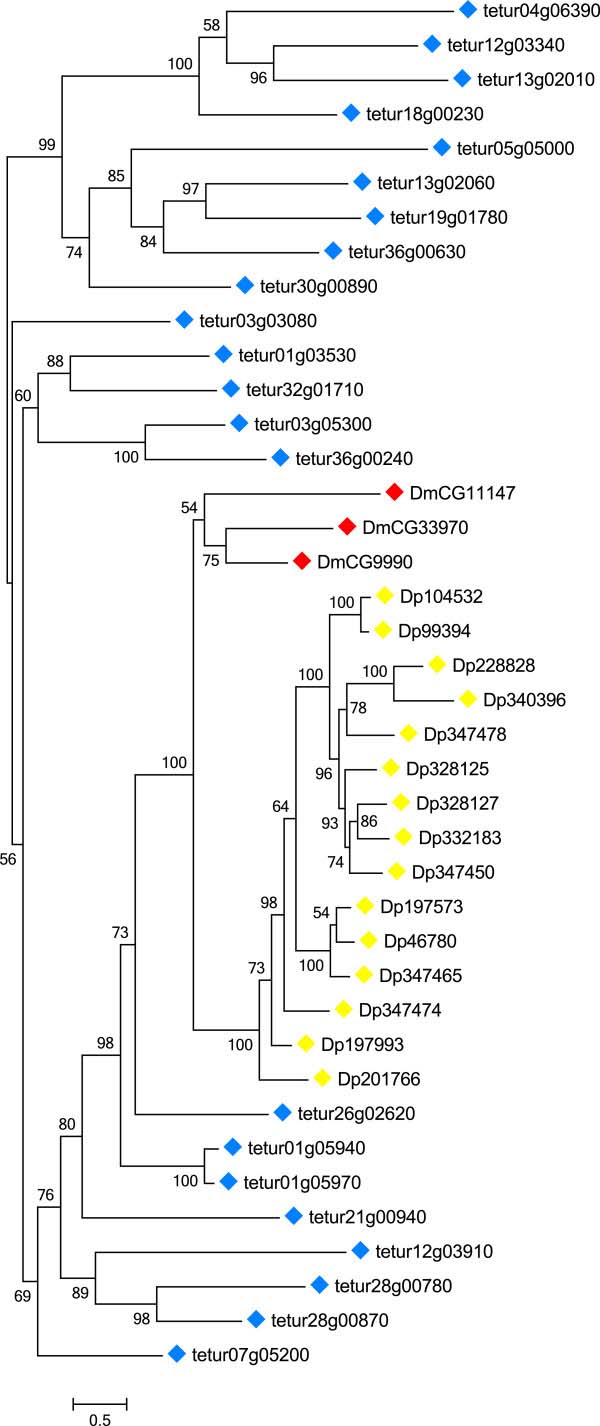
Phylogenetic analysis of ABCH proteins of five metazoan species. See the legend of Figure 2 for procedure and display details.

**The ABCH** subfamily was first discovered in *D*. *melanogaster* and is lacking in mammals, plants or fungi [1,15,47]. In addition to arthropods ([1,8,9,11], this study), members of this subfamily have also been reported in teleost fish [6,7,10]. Most insects have only 3 ABCH genes, while 15 and 22 are present in *D*. *pulex* and *T*. *urticae*, respectively ([9,13], Table 2). Liu et al. [9] suggested that all insect ABCHs diversified from a common ancestral copy. According to our phylogenetic analysis, this insect ancestor seems not to be shared with *T*. *urticae* ABCH proteins (Figure 5). *Tetranychus* ABCHs clustered, similar to *D*. *pulex* ABCHs (see also [11]), into a distinct clade, indicating that the diversity of the ABCH family in *T*. *urticae* has been due to lineage specific duplications. Interestingly, 16 of the 22 *T*. *urticae* ABCHs appear to be intronless (Additional file 3). Although ABCHs have the same structural organization as metazoan ABCGs (HTs, NBD at N-terminal side of TMD), their physiological functions have remained enigmatic. In the zebrafish, *D*. *rerio*, ABCH1 has highest expression in brain, gills and kidney followed by lower expression in intestine, gonads, skeletal muscle and liver [10]. *D*. *melanogaster* ABCHs are enriched in the adult crop and hindgut [113] and at least one of them (CG9990) is glycosylated as shown by mass spectrometry of *N*-glycosylated peptides [52]. An RNAi screen of *D*. *melanogaster* genes revealed that an RNAi line that silences *CG9990* is lethal [114,115]. In addition, microarray analysis demonstrated an almost two-fold upregulation of a *D*. *melanogaster* ABCH (*CG33970*) after cold hardening of adult fruit flies [116]. In the diamondback moth *Plutella xylostella*, it was recently found that an ABCH transporter (Px014955, [117]) was the most up-regulated ABC gene in two resistant strains [13]. The most groundbreaking finding about insect ABCH function was just recently reported by the excellent study of Broehan et al. [8]. RNAi-mediated knockdown of an ABCH gene (*TcABCH*-*9C*) in *T*. *castaneum* larvae resulted in dessication and 100% mortality. Injection of *TcABCH*-*9C* dsRNA into adults also drastically reduced the number of eggs laid and all eggs failed to hatch. Furthermore, cryosections of *TcABCH*-*9C* dsRNA injected larvae stained with Nile Red (a fluorescent dye that stains lipids) revealed a lack of lipids in the epicuticle. Based on these results, the authors suggested that TcABCH-9C functions as a transporter of lipids to the cuticle and is required for the formation of a waterproof barrier in the epicuticle.

### Expression profiling of ABC genes

We assessed expression of ABC genes across development in the *T*. *urticae* London reference strain, as well as in London after transfer from a benign host (bean, *Phaseolus vulgaris*) to two more challenging hosts (*Arabidopsis thaliana* and tomato, *Solanum lycopersicum*; [37]). For the developmental and host transfer experiments, we used existing RNA-seq reads, but we recalculated gene expression using newly described or corrected ABC gene models curated as part of this study. We further examined previously published microarray data to assess the expression profiles of ABC genes in two spider mite strains, MR-VP and MAR-AB, that are resistant to multiple pesticides [42].

**Figure 6:**
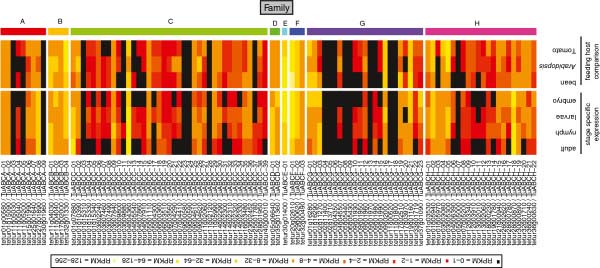
**Heat map of expression values (RPKM values) of 103 *T*. *urticae *ABC genes of mites on different host plants (bean, tomato and *Arabidopsis) *and from four different life stages (embryo, larvae, nymph and adult).** A color legend with corresponding RPKM values is shown at the top of the figure. ABC genes were considered as being expressed when they had an RPKM of >1 in at least one of the spider mite life stages or on one of the plant hosts.

As assessed by RNA-seq expression quantification, the majority of ABC genes were found to be expressed – 88 of the 103 full length *T*. *urticae* ABC genes had an RPKM of >1 in at least one of the spider mite life stages or on one of the plant hosts (Figure 6). In contrast, nearly all *T*. *urticae* ABC fragments or pseudogenes were not expressed (Additional file 8). Most full-length *T*. *urticae* ABC genes for which we detected no expression across development or on different hosts belonged to either ABCA, C or G subfamilies, many of which are tandem duplicated genes ((Table 1), Figure 6). In *C*. *elegans*, tandem duplicated genes were shown to be subfunctionalized, with strong stage or tissue dependent expression [118]. Whether *T*. *urticae* ABC genes that lacked expression support in the existing data are expressed at low levels, in highly restricted expression domains, or alternatively are expressed under specific environmental conditions (i.e., host plants not included in this analysis), remains to be determined.

Many *T*. *urticae* ABC genes were broadly expressed, and more than half (57 genes, or 55% of the total) were expressed across all developmental stages analyzed (embryos, larvae, nymphs, and adults). However, the expression of many ABCC and ABCH genes was restricted (or at their highest levels) in larvae and nymphs; overall, embryos and adult females had the highest number of non-expressed ABCs (40 and 36 ABC genes in the adult and embryos, respectively (Figure 6)). Furthermore, *tetur30g01400* and *tetur20g02610*, members of the ABCE and ABCF subfamilies respectively, showed very high expression in all stages (Figure 6), coinciding with their presumed conserved role in translation regulation (see above). Similar high expression of the *T*. *castaneum* ABCE gene was reported in all developmental stages examined [8]. Finally, within the other ABC subfamilies, *tetur27g01890* (ABCA), *tetur11g04030* (ABCB), *tetur04g04360* (ABCC), *tetur05g06640* (ABCD), *tetur02g11270* (ABCG) and *tetur28g00870* (ABCH) had the highest average expression across developmental stages (Figure 6).

**Figure 7:**
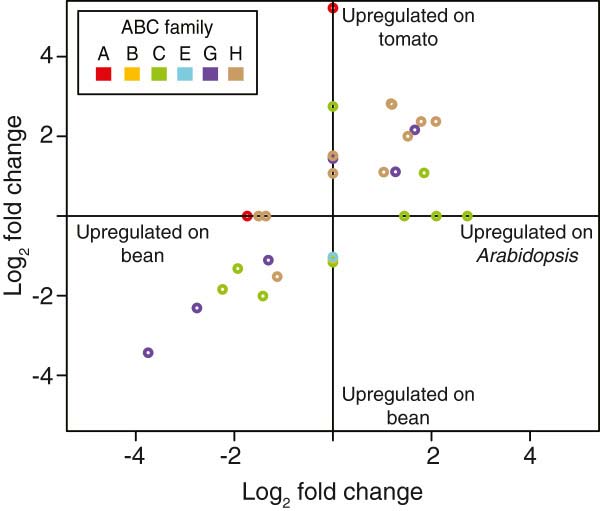
**Full length differentially expressed (fold change ≥ **2**, FDR adjusted p-value <0.05) ABC genes in mites after host plant change from bean to either *Arabidopsis *or tomato.** Subfamilies are color-coded as follows: ABCA, red; ABCB, yellow; ABCC, green; ABCE, blue; ABCG, purple; and ABCH, brown. Genes found to be significant in only one of the pairwise comparisons have had their fold change values assigned to zero for the non-significant comparison. Fold change values of differentially expressed ABC genes can be found in Additional file 13.

Upon host transfer, 22 and 28 ABC genes were differentially expressed in strain London mites transferred from bean to *Arabidopsis* and tomato plants, respectively (Figure 7; fold change ≥ 2, FDR < 0.05 as calculated for all *T*. *urticae* genes, see Methods). We found that 73% (16/22) of the differentially expressed ABC genes in mites transferred to *Arabidopsis* were also differentially expressed on tomato, and belonged to three subfamilies, ABCC (4), -G (5) and -H (7). Surprisingly, ABC genes differentially expressed in two multi-pesticide resistant *T*. *urticae* strains as detected with expression microarrays [42] belonged to the same ABC subfamilies as identified with RNA-seq data in the host transfer experiment (Table 3). The ABCC subfamily has been frequently linked with xenobiotic detoxification in arthropods (see above). On the other hand, arthropod members of the ABCG and H subfamilies have only very recently been reported to be associated with detoxification of xenobiotic compounds (see discussions for the ABCG and ABCH families). The differential expression of members from these ABC subfamilies (G and H) upon host transfer/exposure to xenobiotics should be further (functionally) validated in future studies.

**Table 3:** **Fold changes of full length differentially expressed ABC genes of two multi**-**resistant strains** (**MR**-**VP and MAR**-**AB**) **compared to a susceptible strain** (**London**)

**Tetur ID**	**Name**	**MAR**-**AB**	**MR**-**VP**
tetur01g10390	TuABCC-02	2.87	
tetur03g07460	TuABCC-07	2.03	
tetur03g07490	TuABCC-08	2.03	
tetur03g09800	TuABCC-10	2.13	
tetur03g09880	TuABCC-11	2.04	
tetur04g05540	TuABCC-13		−2.29
tetur05g04300	TuABCC-17	2.45	
tetur40g00010	TuABCC-39	2.00	
tetur18g00230	TuABCH-13		2.21
tetur21g00940	TuABCH-15		2.19

It is worth noting that the differentially expressed *T*. *urticae* ABCC, -G and -H genes mentioned above were not among the most highly differentially expressed genes in these experiments, both in expression level and fold change. In total, 893 and 977 differentially expressed genes were identified in the two multi-resistant strains [42]. Likewise, 2,502 and 3,951 differentially expressed genes were detected in mites when fed on *Arabidopsis* and tomato in comparison with bean, respectively [37]. The Major Facilitator Superfamily, another large and widespread transporter family [5], showed overall a more pronounced response both in the number of genes differentially expressed, and in the fold change values of differentially expressed genes in both resistant strains and after host plant change [37,42]. Thus, despite the exceptional number of ABC transporters in the *T*. *urticae* genome, other transporters and non-transporter proteins also play key roles in the detoxification of xenobiotics [37,42].

## Conclusions

The spider mite *T*. *urticae* is among the most polyphagous pests worldwide and is notorious for its ability to develop resistance against numerous pesticides. One of the prerequisites to study xenobiotic metabolism (pesticides and plant secondary metabolites) in this species is to inventory genes related to detoxification. Here, we provide a survey of the ABC gene superfamily, whose members have frequently been reported to play roles in detoxification, either by directly transporting toxicants out of cells, or after conjugation with glutathione. We identified 103 ABC genes (distributed over eight subfamilies, ABCA-H) in the genome of the spider mite *T*. *urticae*. To date, this is the largest number of ABC genes reported in any metazoan species. The large number is mainly due to lineage-specific expansions in subfamilies C, G and H. Of particular note, most of the differentially expressed ABC genes in acaricide resistant strains and after introduction of mites to challenging host plants belong to these expanded ABC subfamilies. This hints at their potential role in detoxification and may explain their retention after duplication in the mite genome. However, obtaining functional evidence that members of these ABC subfamilies contribute to xenobiotic tolerance should be the priority of further research.

Due to the lineage specific expansions in the ABCC, G, and H families, inferring the function of specific *T*. *urticae* ABC family members based on phylogenetic relationships is not straightforward. Nevertheless, we found clear orthologous relationships between some of the *T*. *urticae* ABC proteins and human ABCC10, ABCG5 and ABCG8, the *D*. *melanogaster* sulfonylurea receptor and the ecdysone-regulated transporter E23. Furthermore, we found a high conservation between *T*. *urticae* ABC proteins and members of the ABCB-half transporters and ABCD, -E, and –F subfamilies, which are known to be involved in fundamental processes. To conclude, this study provides the first thorough ABC gene analysis of a polyphagous arthropod herbivore and represents a useful resource for future biochemical and toxicological studies on the role of ABC transporters in the extremely broad host range and development of pesticide resistance of *T*. *urticae*.

## Methods

### Annotation and phylogeny of ABC transporters

ABCs were identified in a similar way as for *D*. *pulex*[11]. Briefly, tBLASTn searches [119] were performed on the *T*. *urticae* genome sequence assembly (version July 2012, available at http://bioinformatics.psb.ugent.be/orcae/overview/Tetur[120]) using the highly conserved nucleotide binding domain (NBD) of *D*. *melanogaster* ABC proteins as queries. One search was carried out per subfamily, using the sequence of the NBD of a representative *D*. *melanogaster* protein (A: CG1718; B: CG3879 (Mdr49); C: CG9270; D: CG12703; E: CG5651; F: CG9330; G: white; H: CG9990). If the *D*. *melanogaster* transporter had two NBDs, the N-terminal domain was used. All hits with an E-value less than e^-4^ were withdrawn for analysis and gene models were refined or created on the basis of homology and RNA-seq support [120]. The NBDs from those *T*. *urticae* gene models encoding complete ABCs (i.e. not lacking one or both vital domains (TMD and NBD)) were extracted using the ScanProsite facility [121] and the Prosite profile PS50893. *T*. *urticae* ABC protein NBDs were aligned with NBDs of *D*. *melanogaster* and human ABC transporters using MUSCLE [122]. Model selection was done with Prottest 2.4 [123]. According to the Akaike information criterion LG+F+G was optimal for phylogenetic analysis (see Additional file 9 and Additional file 10 for protein alignment and likelihood scores, respectively). A maximum likelihood phylogenetic analysis of *T*. *urticae*, *D*. *melanogaster* and human ABC protein NBDs, bootstrapping with 1000 pseudoreplicates, was performed using Treefinder [124] to confirm the position of *T*. *urticae* ABCs within ABC classes (A-G). A similar phylogenetic analysis, restricted to N-terminal NBDs of *T*. *urticae*, was also performed (optimal model according to the Akaike Information Criterion: LG+I+G+F, see Additional file 9 and Additional file 10 for protein alignment and likelihood score, respectively). Similar to previous studies, in the phylogenetic analysis using *T*. *urticae*, *D*. *melanogaster* and human ABC protein NBDs C-terminal NBDs of the ABCC subfamily clustered together with NBDs of the ABCB subfamily [9,45,125]. The subfamily assignment was further confirmed by BLASTp analyses of the manually corrected models on the NCBI website. We adopted the guidelines set forth by the human genome organization nomenclature committee (HGNC) for naming the *T*. *urticae* ABC proteins. Separate phylogenetic analyses on full ABC protein sequences of *T*. *urticae*, *D*. *pulex*, *C*. *elegans*, *D*. *melanogaster* and *H*. *sapiens* ABCs were also carried out for each subfamily, using the same methodology as above (for ABC protein alignments see Additional file 9, for model choice and likelihood scores see Additional file 10). According to previous studies [7,11], this approach facilitates bioinformatics analyses and results in a more meaningful degree of resolution in phylogenetic analysis. Finally, in order to detect ABC pseudogenes/fragments not containing ABC NBDs, all protein sequences of complete ABCs were used as query in tBLASTn-searches against the *T*. *urticae* genome. Phylogenetic trees were visualized and edited using MEGA5 [126] and CorelDraw X3 (Corel Inc.), respectively.

### Sequence similarity, transmembrane prediction and gene structure of T. *urticae* ABC proteins

ABC protein sequence similarities and identities were calculated using MatGAT 2.03 [127] using default settings (BLOSUM50 matrix, gap opening and extending gap penalty set to 12 and 2, respectively). Transmembrane domains of *T*. *urticae* ABCs were predicted using the SCAMPI prediction server [128]. Subcellular localization was predicted using TargetP 1.0 [129] . Gene structures of *T*. *urticae* ABCs were visualized using the coordinates of each *T*. *urticae* ABC transporter (available at [120]) and the fancyGene visualization software [130]. N-glycosylation sites were predicted with NetNGlyc1.0 server [131]. Only N-glycosylation sites with a “potential” score > 0.5 and with a jury agreement (“++”-sign or higher) were included in analyses. O-glycosylation sites were predicted using NetOGlyc 3.1 server [132]. If the G-score was higher than 0.5 the residue was considered to be O-glycosylated. The number of O-glycoslated sites (glycosylated serines and threonines) is shown in Table 1).

### Expression profiling of ABC genes

Expression profiling of ABC genes was assessed using microarray expression data of two multi-pesticide resistant strains (MR-VP and MAR-AB) [42] and a previously published RNA-seq dataset [37]. The RNA-seq dataset consists of replicated RNA-seq libraries of spider mites feeding on different host plants (bean, tomato and *Arabidopsis*) and a single RNA-seq library for different developmental stages of spider mites (embryo, larvae, nymph and adult). Experimental details can be found in Grbić et al. [37] and the RNA-seq data are available via Gene Expression Omnibus under reference GSE32342. To ensure the best possible alignment of RNA-seq reads to our manually curated ABC transporter gene models, we re-mapped the RNA-seq reads to the spider mite genome with an updated annotation (Nov 15, 2012 release; [120]). Read alignments and expression quantification were performed after Grbić et al. [37]. For host transfer experiments, differential gene expression was assessed with the DESeq R package [133] as previously described [37]. For the microarray experiment, differentially expressed genes were assessed as reported earlier [42]. For both the host transfer experiment (RNA-seq) and expression profiling with multi-pesticide resistant strains (microarrays), ABC genes with a fold change higher than two and a FDR adjusted p-value less than 0.05 were considered as differentially expressed.

## Abbreviations

ABC: ATP-binding cassette; FDR: False discovery rate; FT: Full transporter; GST: Glutathione S-transferase; HT: Half transporter; MDR: Multidrug resistance protein; MRP: Multidrug resistance associated protein; P-gp: P-glycoprotein; RPKM: Reads per kilobase per million mapped reads; SUR: Sulfonylurea receptor

## Competing interest

The authors declare that they have no competing interests.

## Authors’ contributions

WD and TVL designed research. WD and EJO analyzed data. WD and TVL wrote the manuscript, with input from RMC, EJO and LT. All authors read and approved the final manuscript.

## Supplementary Material

Additional file 1 **Midpoint rooted maximum likelihood phylogenetic tree of ABC NBDs of *D. melanogaster*, *H. sapiens *and *T. urticae*.** For amino acid alignment, amino acid substitution model and likelihood score of the constructed phylogenetic tree see Additional file 9 and Additional file 10. Main nodes were collapsed to create a better overview of the phylogenetic relationships between the different ABC subfamilies. Numbers at the branch point of each node represent the bootstrap value resulting from 1000 pseudoreplicates (LR-ELW). The scale bar represents 0.5 amino-acid substitutions per site. For accession numbers of metazoan ABC protein sequences see Additional file 11 while *T. urticae* ABC protein sequences can be found in Additional file 12. Click here for file

Additional file 2 ***T. urticae *** **ABC fragments.** Click here for file

Additional file 3 **Exon-intron pattern of 103 *T. urticae *ABC genes.** Exons are depicted as light grey boxes while strandlines represent introns. Within exons red boxes represent NBDs, while small dark grey boxes represent transmembrane helices (TM). Solid arrows indicate direction of transcription. Click here for file

Additional file 4 **Phylogenetic analysis of ABCA proteins of five metazoan species, derived according to the procedure in the Figure** 2**legend.** Click here for file

Additional file 5 **Identity/similarity matrices between *T. urticae, D. pulex, D. melanogaster *and *H. sapiens *ABC proteins.** In the upper triangle identity values are shown, while in the lower triangle similarity values are presented. Click here for file

Additional file 6 **Phylogenetic analysis of ABCD proteins of five metazoan species, derived according to the procedure in the Figure** 2**legend.** Click here for file

Additional file 7 **Phylogenetic analysis of ABCE and ABCF proteins of five metazoan species, derived according to the procedure in the Figure** 2**legend.** Click here for file

Additional file 8 **Heat plot of mean expression values (rpkm) of *T. urticae *** **ABC fragments from mites on different host plants (bean, tomato and *Arabidopsis*) and of expression values of *T. urticae *** **ABC fragments from four different life stages (embryo, larvae, nymph and adult).** Click here for file

Additional file 9 **Alignment of N-terminal NBDs of *T. urticae *ABC proteins (ABC_N-term_NBD_Tetranychus.fas), N- and C-terminal NBDs of metazoan ABC proteins (ABC_NBD_Metazoa_simple.fas) and of full length metazoan ABCA, -B, C, D, E, F, G and H protein sequences (ABCA-H.fas) (bundled in a .rar file) (examination of the files in this .rar file requires the BioEdit program** (http://www.mbio.ncsu.edu/bioedit/bioedit.html)). Click here for file

Additional file 10 Amino acid substitution models used for maximum likelihood phylogenetic analyses and likelihood scores of constructed phylogenetic trees. Click here for file

Additional file 11 Accession numbers of sequences used for phylogenetic analysis. Click here for file

Additional file 12 **103 *T. urticae *ABC protein sequences (.fasta) (examination of this file requires the BioEdit program** (http://www.mbio.ncsu.edu/bioedit/bioedit.html)). Click here for file

Additional file 13 **Fold change values of differentially expressed ABC genes of mites after host plant change to either *Arabidopsis *** **or tomato.** Click here for file
