# Supplementary figures and images for: A burst of ABC genes in the genome of the polyphagous spider mite Tetranychus urticae
Source: BMC Genomics. 2013 May 10;14:317. doi: 10.1186/1471-2164-14-317 (PMC3724490; doi:10.1186/1471-2164-14-317)

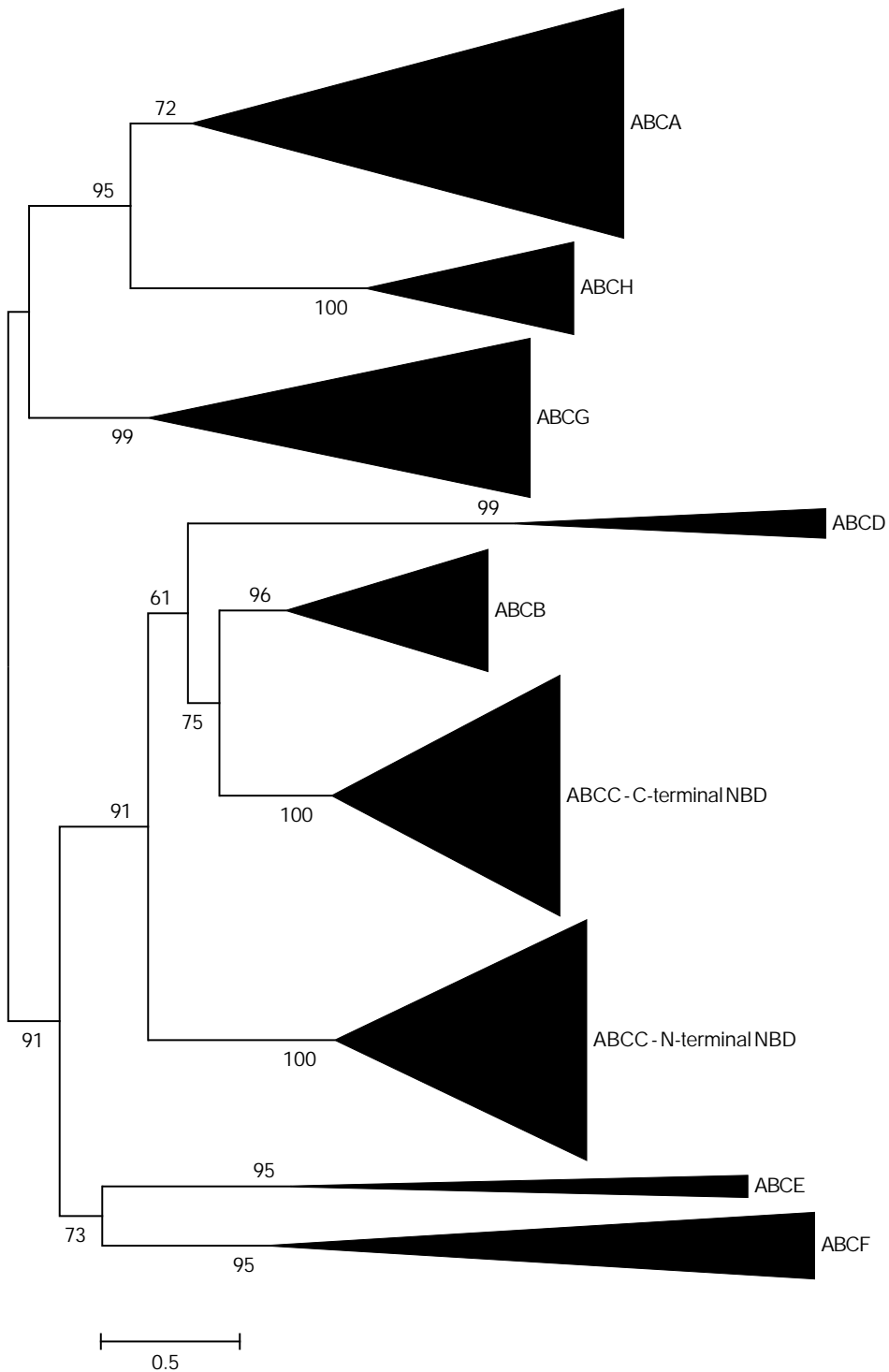

Supplement: Additional file 1 — Midpoint rooted maximum likelihood phylogenetic tree of ABC NBDs of D. melanogaster, H. sapiens and T. urticae. For amino acid alignment, amino acid substitution model and likelihood score of the constructed phylogenetic tree see Additional file 9 and Additional file 10. Main nodes were collapsed to create a better overview of the phylogenetic relationships between the different ABC subfamilies. Numbers at the branch point of each node represent the bootstrap value resulting from 1000 pseudoreplicates (LR-ELW). The scale bar represents 0.5 amino-acid substitutions per site. For accession numbers of metazoan ABC protein sequences see Additional file 11 while T. urticae ABC protein sequences can be found in Additional file 12. [file 1471-2164-14-317-S1.pdf]

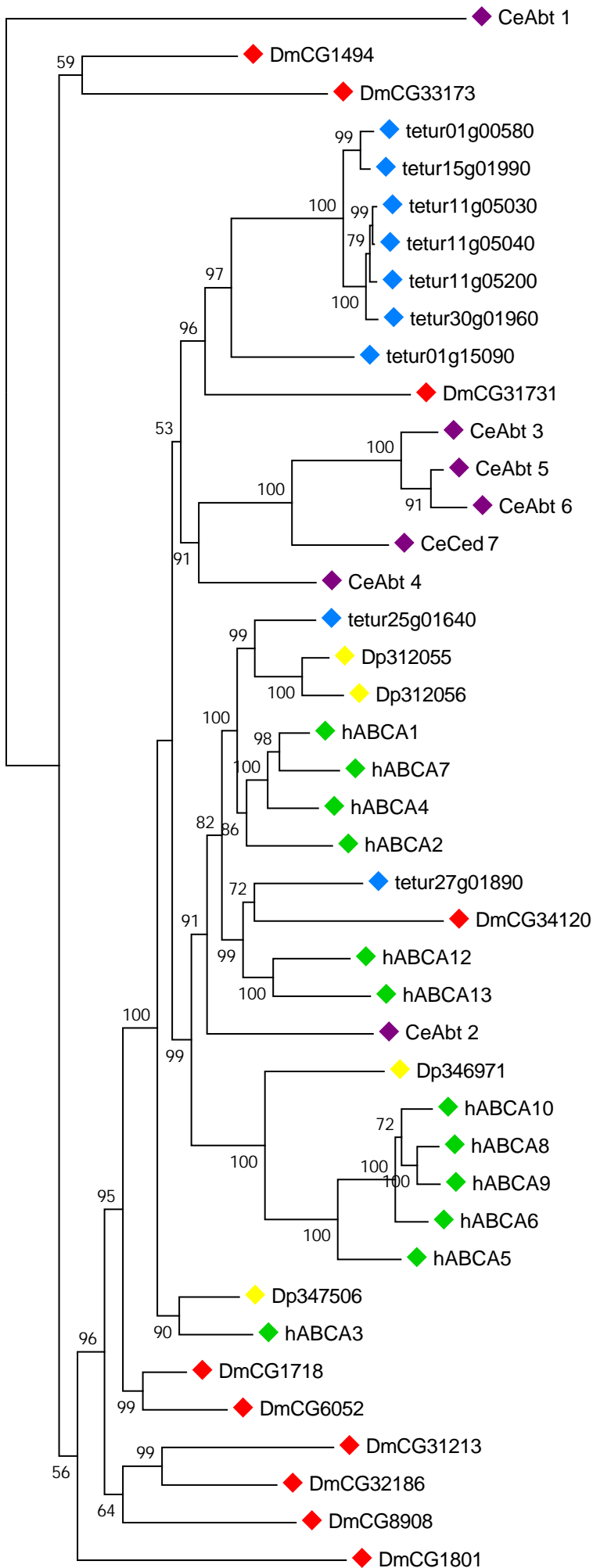

0.5

Supplement: Additional file 4 — Phylogenetic analysis of ABCA proteins of five metazoan species, derived according to the procedure in the Figure 2 legend. [file 1471-2164-14-317-S4.pdf]

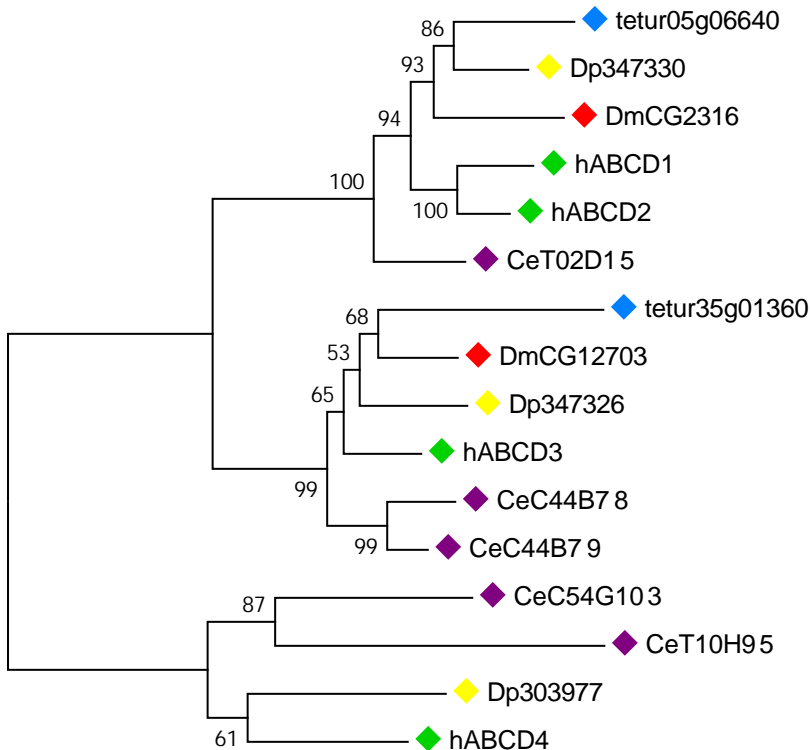

Supplement: Additional file 6 — Phylogenetic analysis of ABCD proteins of five metazoan species, derived according to the procedure in the Figure 2 legend. [file 1471-2164-14-317-S6.pdf]

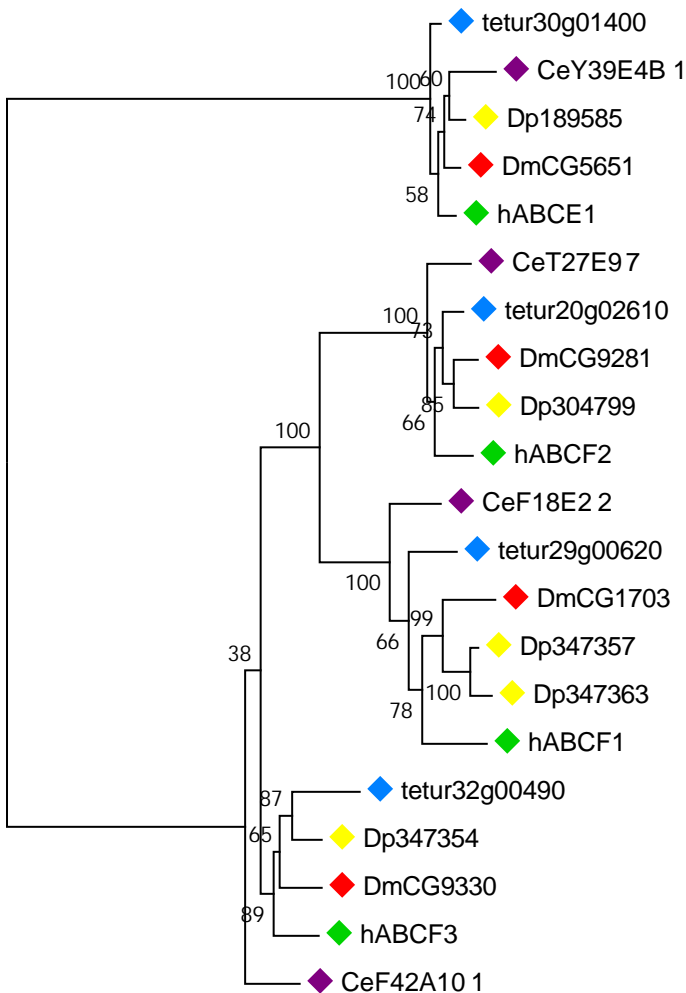

0.5

Supplement: Additional file 7 — Phylogenetic analysis of ABCE and ABCF proteins of five metazoan species, derived according to the procedure in the Figure 2 legend. [file 1471-2164-14-317-S7.pdf]

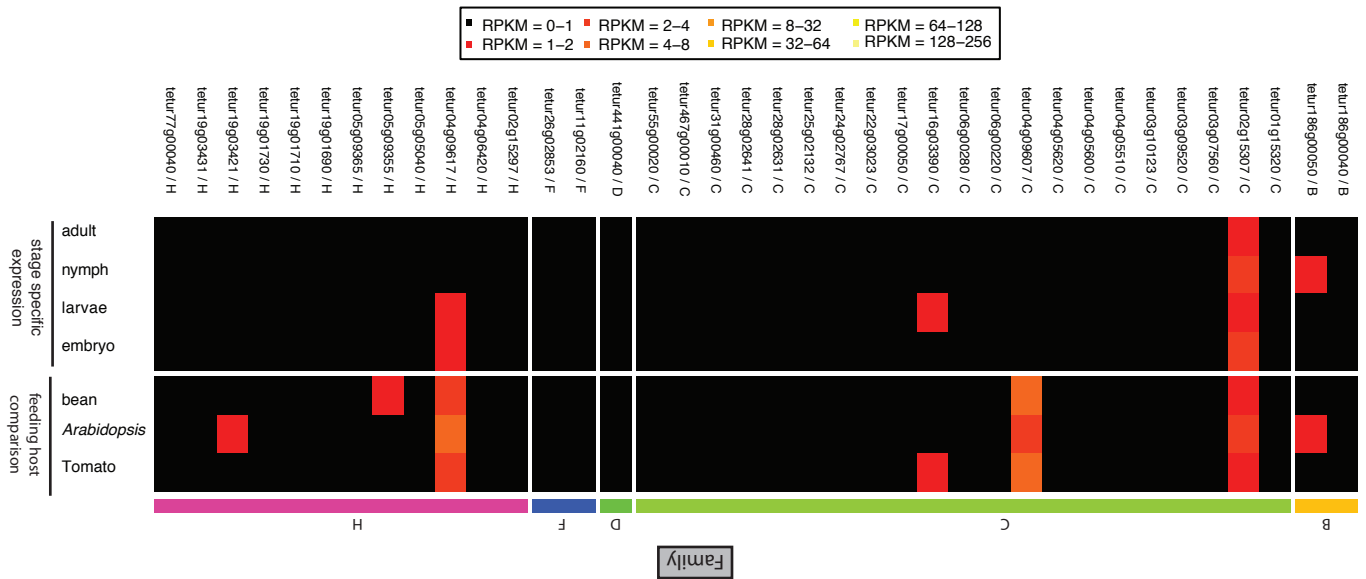

Supplement: Additional file 8 — Heat plot of mean expression values (rpkm) of T. urticae ABC fragments from mites on different host plants (bean, tomato and Arabidopsis ) and of expression values of T. urticae ABC fragments from four different life stages (embryo, larvae, nymph and adult). [file 1471-2164-14-317-S8.pdf]
