# Supplementary material for: A burst of ABC genes in the genome of the polyphagous spider mite Tetranychus urticae
Source: BMC Genomics. 2013 May 10;14:317. doi: 10.1186/1471-2164-14-317 (PMC3724490; doi:10.1186/1471-2164-14-317)
Supplement: Additional file 2 — T. urticae ABC fragments. [file 1471-2164-14-317-S2.docx]

| **ABC-transporter fragments** | **Length (AA)** | **best BLASTp hit with complete ABC transporter** | **ABC subfamily** | **E-Value** |  |
| --- | --- | --- | --- | --- | --- |
| tetur186g00040 | 112 | tetur11g04030 | B | 1,00E-73 |  |
| tetur186g00050 | 161 | tetur11g04030 | B | 1,00E-54 |  |
| tetur01g15320 | 129 | tetur01g15330 | C | 2,00E-64 |  |
| tetur02g15307 | 32 | tetur09g00590 | C | 6,00E-06 |  |
| tetur03g07560 | 310 | tetur03g07490 | C | 7,00E-61 |  |
| tetur03g09520 | 171 | tetur03g09880 | C | 8,00E-50 |  |
| tetur03g10123 | 78 | tetur09g04620 | C | 2,00E-05 |  |
| tetur03g10133 | 28 | tetur03g09880 | C | 4,00E-09 |  |
| tetur04g05510 | 196 | tetur04g05540 | C | 8,00E-53 |  |
| tetur04g05600 | 60 | tetur04g05540 | C | 1,00E-19 |  |
| tetur04g05620 | 234 | tetur04g05540 | C | 1,00E-56 |  |
| tetur04g09607 | 60 | tetur04g05540 | C | 3,00E-16 |  |
| tetur06g00220 | 693 | tetur06g00360 | C | 0.0 |  |
| tetur06g00280 | 941 | tetur06g00360 | C | 0.0 |  |
| tetur16g03390 | 119 | tetur16g03480 | C | 2,00E-20 |  |
| tetur17g00050 | 83 | tetur14g02290 | C | 5,00E-11 |  |
| tetur22g03023 | 68 | tetur25g01780 | C | 6,00E-17 |  |
| tetur24g02767 | 66 | tetur14g02310 | C | 1,00E-05 |  |
| tetur25g02132 | 36 | tetur25g01780 | C | 4,00E-22 |  |
| tetur28g02631 | 52 | tetur01g07880 | C | 1,00E-17 |  |
| tetur28g02641 | 168 | tetur01g07880 | C | 2,00E-20 |  |
| tetur31g00460 | 174 | tetur25g01780 | C | 6,00E-24 |  |
| tetur467g00010 | 177 | tetur04g04360 | C | 1,00E-79 |  |
| tetur55g00020 | 202 | tetur01g07880 | C | 5,00E-31 |  |
| tetur441g00040 | 45 | tetur35g01360 | D | 3,00E-25 |  |
| tetur11g02160 | 464 | tetur32g00490 | F | 5,00E-175 |  |
| tetur26g02853 | 33 | tetur32g00490 | F | 4,00E-05 |  |
| tetur02g15297 | 40 | tetur04g06390 | H | 6,00E-11 |  |
| tetur04g06420 | 158 | tetur04g06390 | H | 5,00E-65 |  |
| tetur04g09617 | 52 | tetur18g00230 | H | 9,00E-16 |  |
| tetur05g05040 | 101 | tetur05g05000 | H | 3,00E-18 |  |
| tetur05g09355 | 66 | tetur05g05000 | H | 5,00E-36 |  |
| tetur05g09365 | 92 | tetur05g05000 | H | 5,00E-46 |  |
| tetur19g01690 | 152 | tetur19g01780 | H | 4,00E-72 |  |
| tetur19g01710 | 297 | tetur19g01780 | H | 1,00E-70 |  |
| tetur19g01730 | 218 | tetur19g01780 | H | 1,00E-151 |  |
| tetur19g03421 | 56 | tetur19g01780 | H | 3,00E-18 |  |
| tetur19g03431 | 144 | tetur19g01780 | H | 3,00E-64 |  |
| tetur77g00040 | 195 | tetur04g06390 | H | 1,00E-86 |  |
| scaffold 30 : 884878..884940 * | 21 | tetur30g01960 | A | 2,00E-06 |  |
| scaffold 266: 382..438, 441..512* | 19 | tetur04g05540 | C | 2,00E-13 |  |
| scaffold 211: 25-141* | 39 | tetur04g05540 | C | 1,00E-16 |  |
| * no gene model could be constructed for these loci | | | |  |  |
