# Supplementary material for: A burst of ABC genes in the genome of the polyphagous spider mite Tetranychus urticae
Source: BMC Genomics. 2013 May 10;14:317. doi: 10.1186/1471-2164-14-317 (PMC3724490; doi:10.1186/1471-2164-14-317)
Supplement: Additional file 3 — Exon-intron pattern of 103 T. urticae ABC genes. Exons are depicted as light grey boxes while strandlines represent introns. Within exons red boxes represent NBDs, while small dark grey boxes represent transmembrane helices (TM). Solid arrows indicate direction of transcription. [file 1471-2164-14-317-S3.pdf]

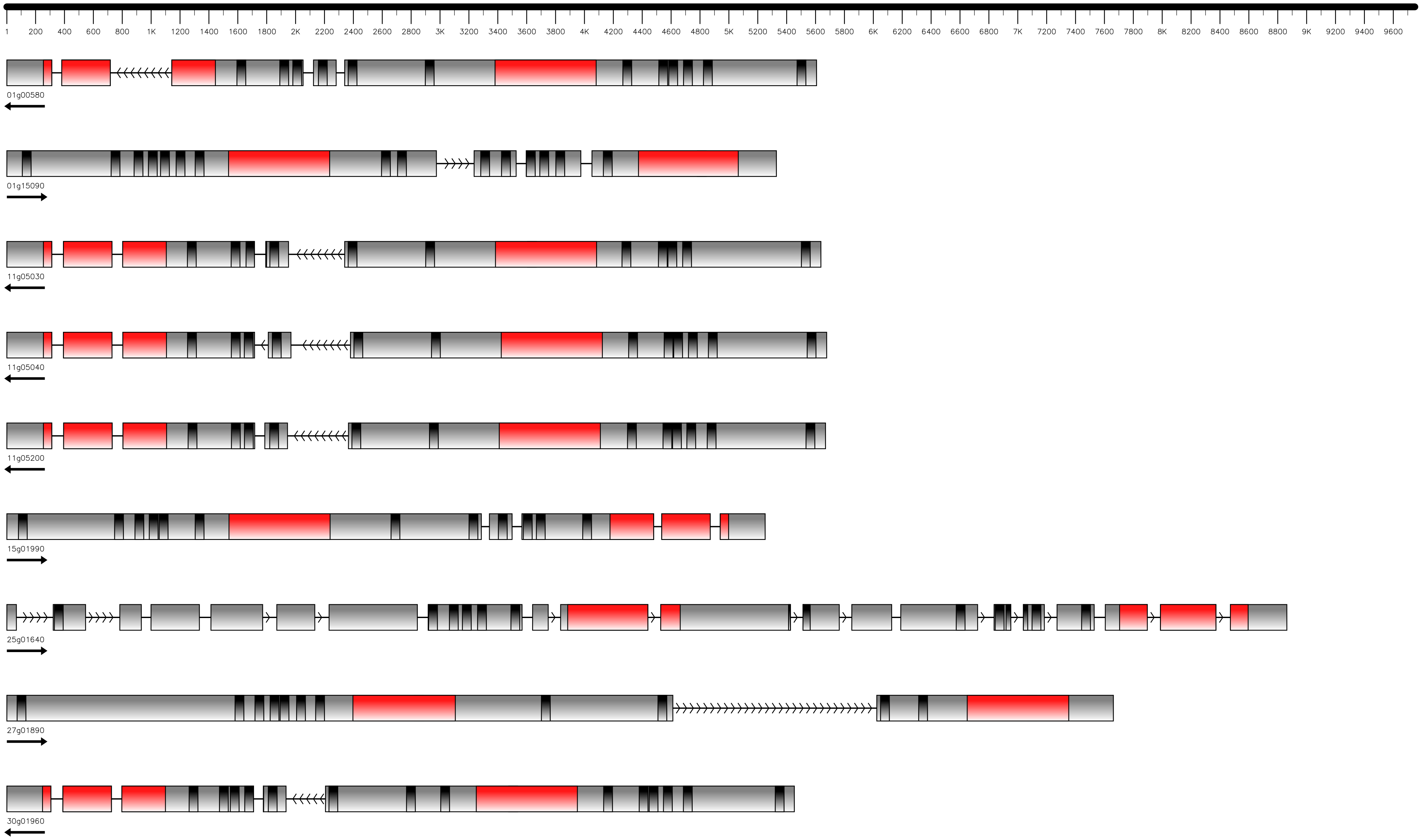

ABCA subfamily

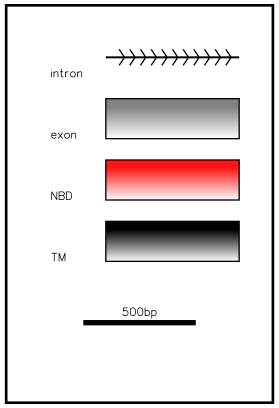

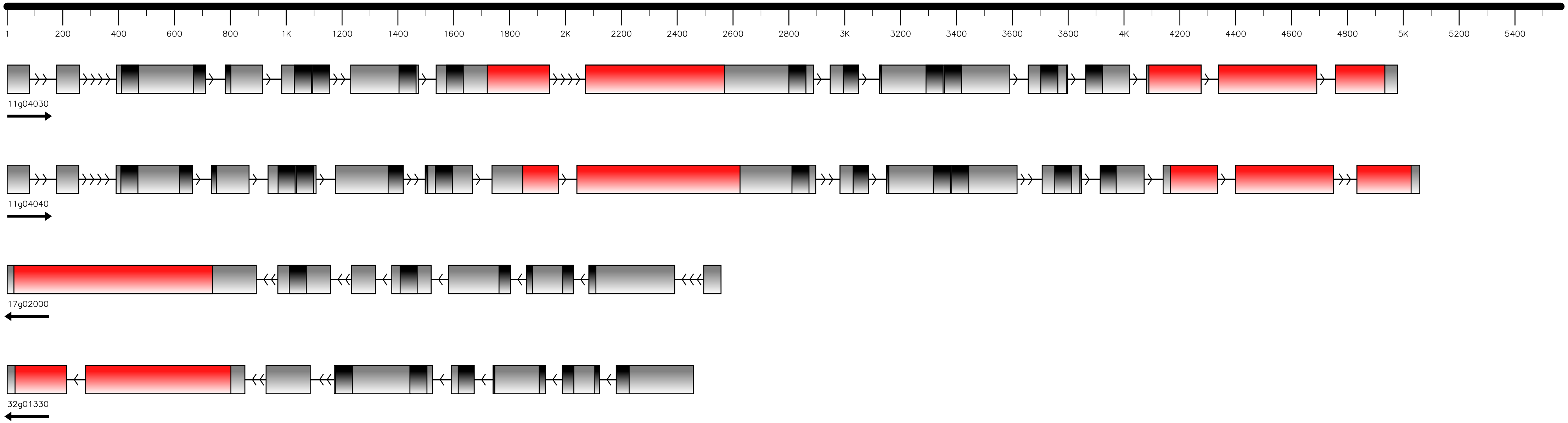

ABCB subfamily

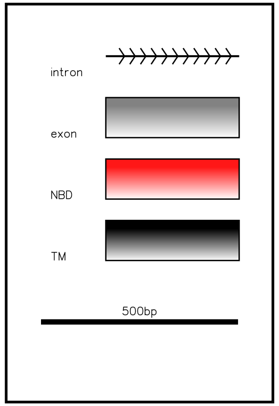

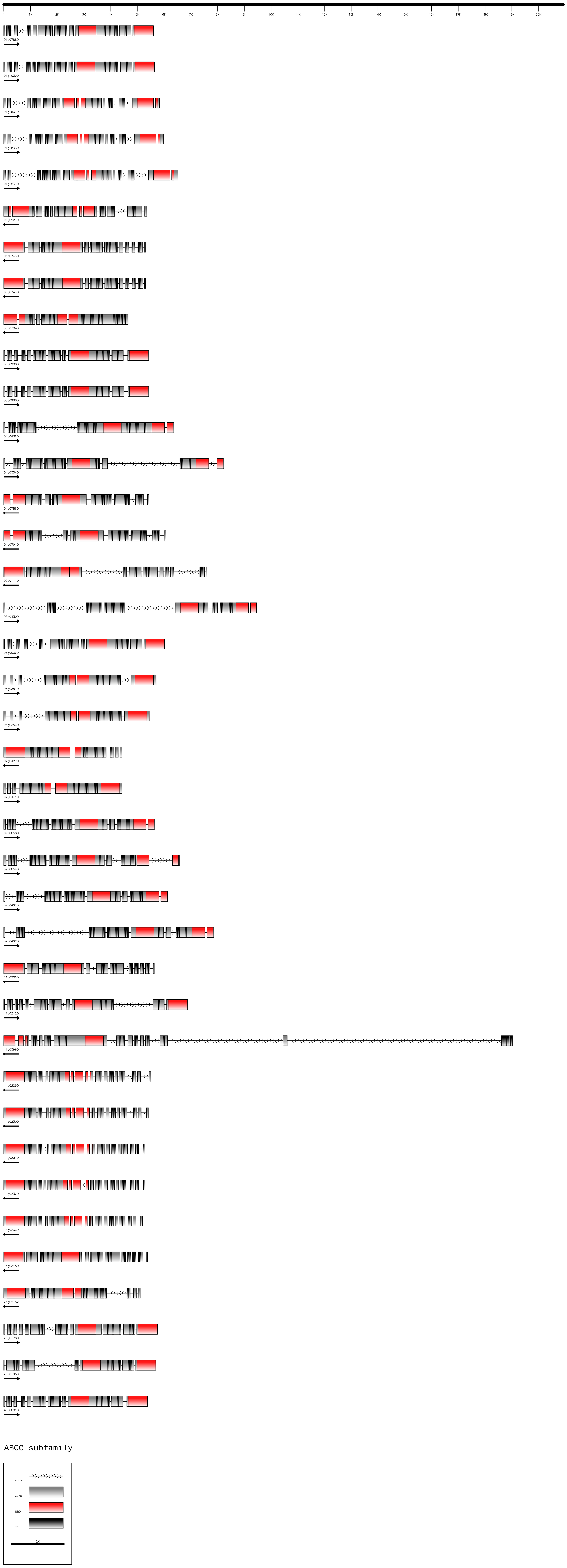
1
1K
2K
3K
4K
5K
6K
7K
8K
9K
10K
11K
12K
13K
14K
15K
16K
17K
18K
19K
20K
01g07880
01g10390
01g15310
01g15330
01g15340
03g02240
03g07460
03g07490
03g07840
03g09800
03g09880
04g04360
04g05540
04g07860
04g07910
05g01110
05g04300
06g00360
06g03510
06g03560
07g04290
07g04410
09g00580
09g00590
09g04610
09g04620
11g02060
11g02120
11g05990
14g02290
14g02300
14g02310
14g02320
14g02330
16g03480
23g02452
25g01780
28g01950
40g00010
ABCC subfamily
intron
exon
NBD
TM
2K

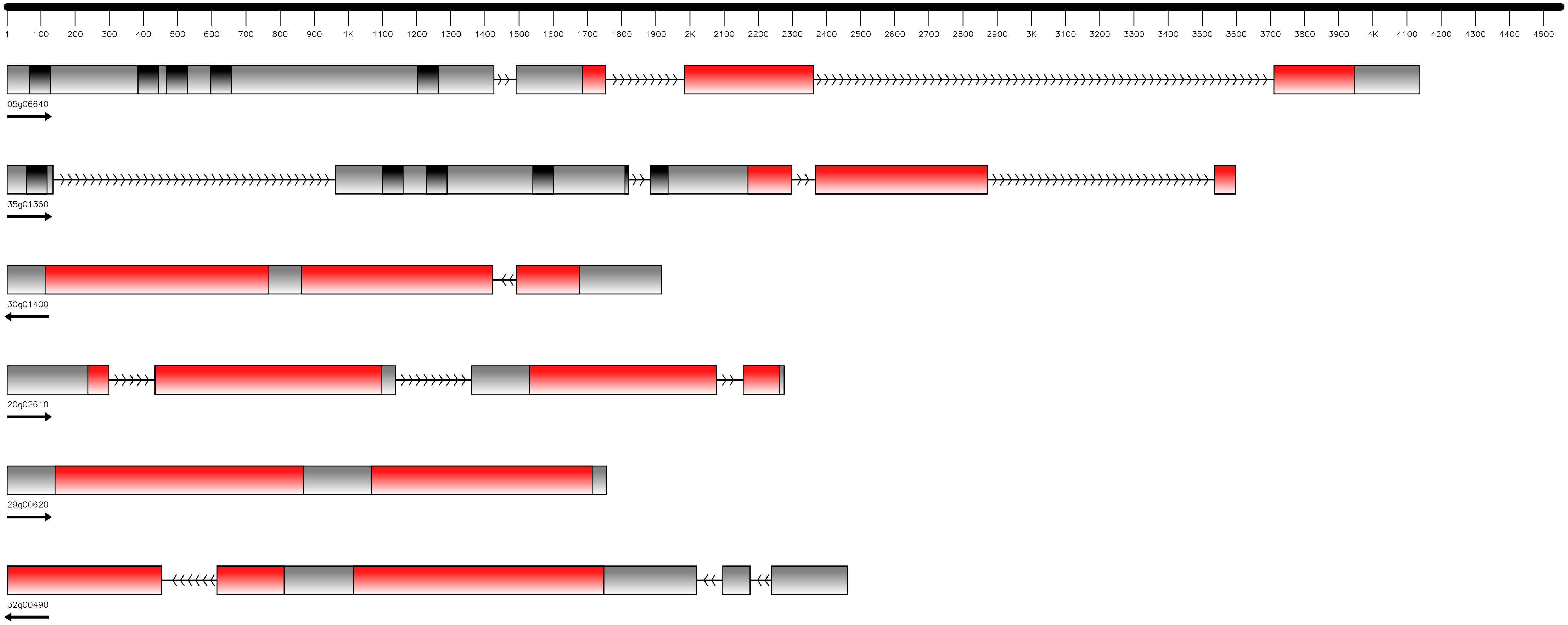

ABCD,E and F subfamily

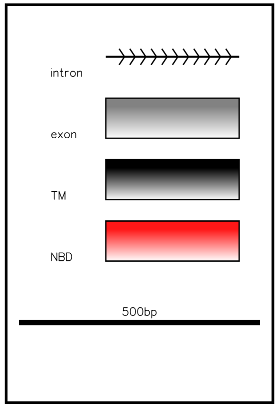

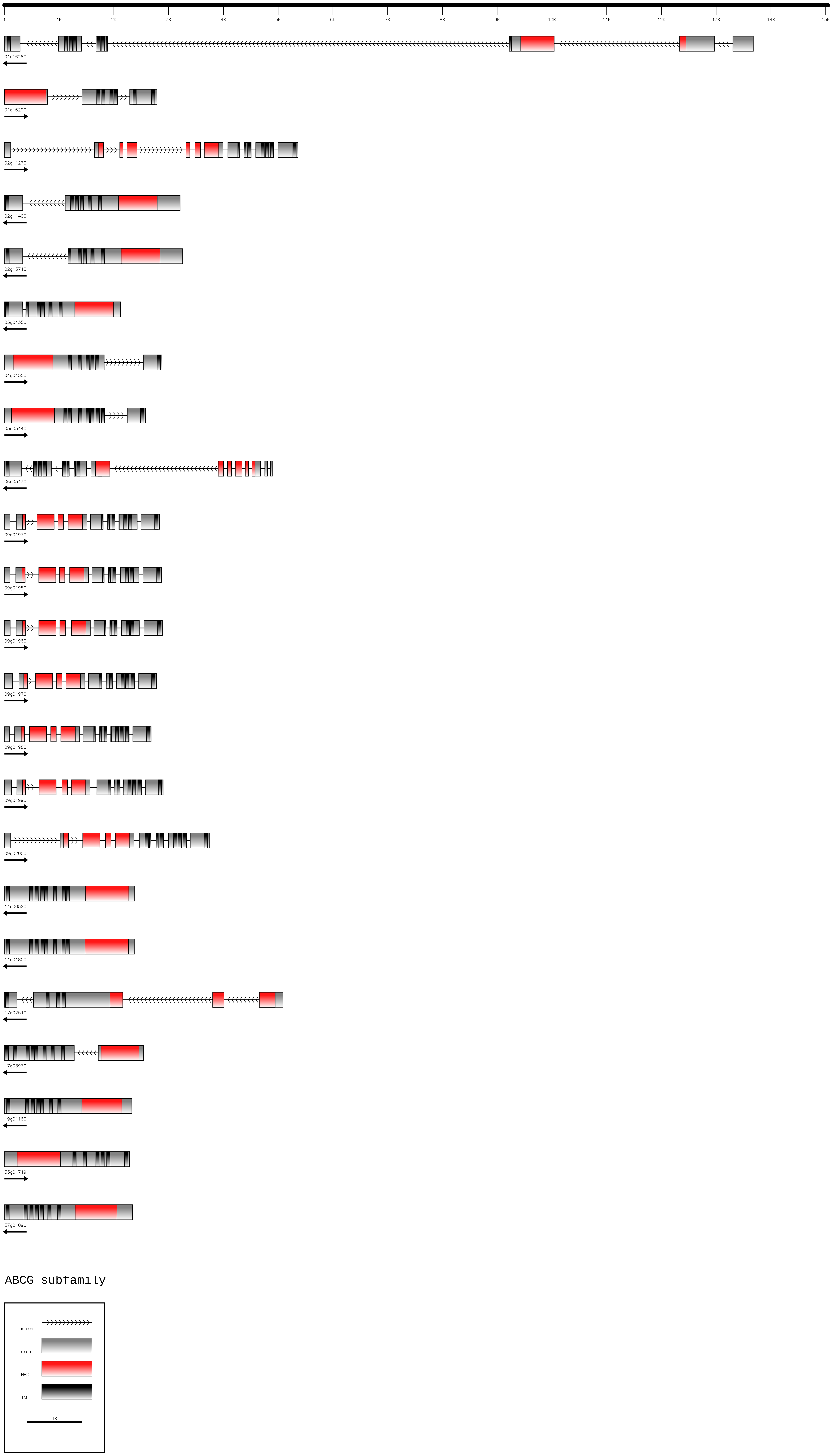

ABCG subfamily

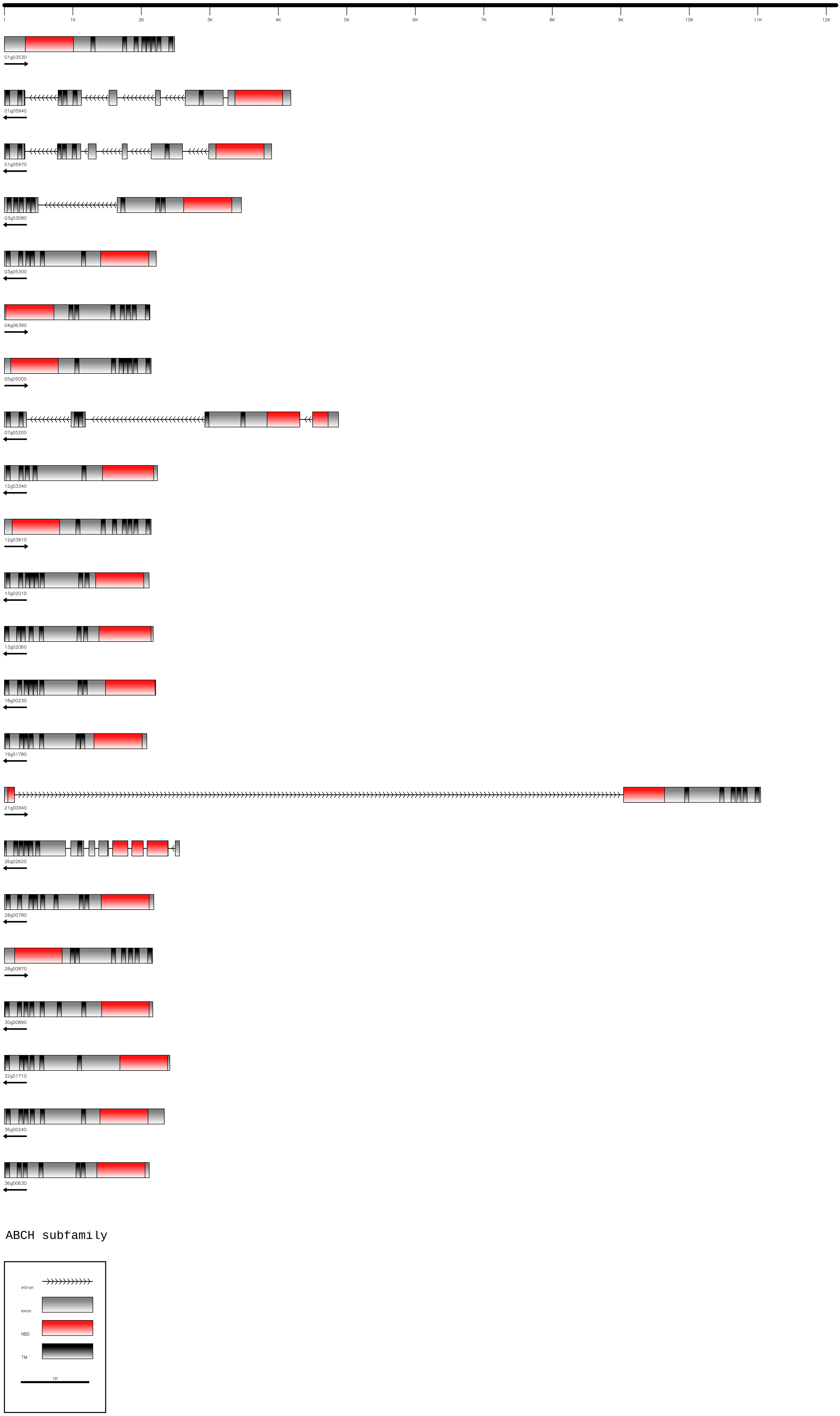
1
1K
2K
3K
4K
5K
6K
7K
8K
9K
10K
11K
12K
01g03530
01g05940
01g05970
03g03080
03g05300
04g06390
05g05000
07g05200
12g03340
12g03910
13g02010
13g02060
18g00230
19g01780
21g00940
26g02620
28g00780
28g00870
30g00890
32g01710
36g00240
36g00630
ABCH subfamily
intron
exon
NBD
TM
1K
